# Supplementary material for: A reinforcement learning and sequential sampling model constrained by gaze data
Source: PLoS Comput Biol. 2026 Mar 6;22(3):e1014052. doi: 10.1371/journal.pcbi.1014052 (PMC12991361; doi:10.1371/journal.pcbi.1014052)
Supplement: S1 Appendix — (PDF) [file pcbi.1014052.s001.pdf]

S1 Appendix: Model descriptions

Here we describe the eight RL-SSMs that were tested. The only difference between them is the linking function that maps learned Q-values and proportional gaze onto the mean drift rates, $v_{i,t}$.

Table 1. Overview of models

Model Number	Model Name	Linking Function
1	Q	$v_{i,t} = \beta_Q \cdot Q_{i,t}$
2	Q*gaze	$v_{i,t} = \beta_Q \cdot Q_{i,t} \cdot gaze_{i,t}$
3	Q + gaze	$v_{i,t} = \beta_Q \cdot Q_{i,t} + \beta_{gaze} \cdot gaze_{i,t}$
4	softmax(Q)	$v_{i,t} = \beta_Q \cdot \frac{\exp(\theta \cdot Q_{i,t})}{\sum_k \exp(\theta \cdot Q_{k,t})}$
5	softmax(Q*gaze)	$v_{i,t} = \beta_Q \cdot \frac{\exp(\theta \cdot Q_{i,t} \cdot gaze_{i,t})}{\sum_k \exp(\theta \cdot Q_{k,t} \cdot gaze_{k,t})}$
6	softmax(Q)*gaze	$v_{i,t} = \beta_Q \cdot \frac{\exp(\theta \cdot Q_{i,t})}{\sum_k \exp(\theta \cdot Q_{k,t})} \cdot gaze_{i,t}$
7	softmax(Q) + gaze	$v_{i,t} = \beta_Q \cdot \frac{\exp(\theta \cdot Q_{i,t})}{\sum_k \exp(\theta \cdot Q_{k,t})} + \beta_{gaze} \cdot gaze_{i,t}$
8	softmax(Q + gaze)	$v_{i,t} = \beta_Q \cdot \frac{\exp\left(\theta \cdot (Q_{i,t} + \beta_{gaze} \cdot gaze_{i,t})\right)}{\sum_k \exp\left(\theta \cdot (Q_{k,t} + \beta_{gaze} \cdot gaze_{k,t})\right)}$

1. Q Model. The first and simplest model that we tested maps Q-values linearly onto mean drift rates and assumes no effect of gaze.

2. Q*gaze Model. The second model assumes a linear relationship between Q-values and mean drift rates, but with a multiplicative effect of gaze. Gaze will have a larger effect on drift rates for options with larger Q-values.

3. Q + gaze Model. The third model assumes a linear relationship between Q-values and mean drift rates with an additive effect of gaze. The effect of gaze is independent of learned value.

4. softmax(Q) Model. In the fourth model, Q-values are non-linearly mapped to mean drift rates via the softmax function. There are no gaze effects. Importantly, the softmax transformation causes mean drift rates to depend only on the *differences* between Q-values, since:

$$v_{i,t} = \beta_Q \cdot \frac{\exp(\theta \cdot Q_{i,t})}{\sum_k \exp(\theta \cdot Q_{k,t})} = \beta_Q \cdot \frac{1}{1 + \sum_{k \neq i} \exp[-\theta \cdot (Q_{i,t} - Q_{k,t})]} \tag{1}$$

5. softmax(Q*gaze) Model. The fifth model assumes a multiplicative gaze effect that operates prior to the softmax transformation. Gaze modulates the cached Q-values directly, and the softmax function normalizes the gaze-modulated Q-values to the unit interval. If neither option is fixated on a particular trial ($gaze_{i,t} = 0$ for all i), the mean drift rate for every option will be $v_{i,t} = \beta_Q / K$, where K denotes the number of available options on that trial. Importantly, this model implies that the softmax normalization must occur at choice time. This is because gaze modulates the inputs to the softmax, and gaze is only measured at the time of choice.

6. softmax(Q)*gaze Model. The sixth model assumes a multiplicative gaze effect that operates after the softmax transformation. If neither option is fixated on a particular trial, the mean drift rate for every option will be $v_{i,t} = 0$, as in Model 2.

7. softmax(Q) + gaze Model. The seventh model assumes a non-linear mapping of Q-values to mean drift rates via the softmax, along with an independent, additive effect of gaze.

8. softmax(Q + gaze) Model. The eighth model may appear to be additive, but the joint effects of gaze and value are multiplicative:

$$\begin{aligned} v_{i,t} &= \beta_Q \cdot \frac{\exp\left(\theta \cdot (Q_{i,t} + \beta_{gaze} \cdot gaze_{i,t})\right)}{\sum_k \exp\left(\theta \cdot (Q_{k,t} + \beta_{gaze} \cdot gaze_{k,t})\right)} \\ &= \beta_Q \cdot \frac{\exp(\theta \cdot Q_{i,t}) \cdot \exp(\gamma \cdot gaze_{i,t})}{\sum_k \exp(\theta \cdot Q_{k,t}) \cdot \exp(\gamma \cdot gaze_{k,t})} \end{aligned} \tag{2}$$

where $\gamma = \theta \cdot \beta_{gaze}$. Even though Q-values and gaze values are added together inside the softmax, their exponentiated values multiply each other. Like Model 5, this model implies that the softmax normalization must occur at choice time, when gaze is measured.
