# Supplementary material for: A reinforcement learning and sequential sampling model constrained by gaze data
Source: PLoS Comput Biol. 2026 Mar 6;22(3):e1014052. doi: 10.1371/journal.pcbi.1014052 (PMC12991361; doi:10.1371/journal.pcbi.1014052)
Supplement: S2 Appendix — (PDF) [file pcbi.1014052.s002.pdf]

S2 Appendix: Trial-dependent decision threshold

Our primary models assume a static decision threshold; that is, that the decision maker requires the same amount of evidence to make a decision on all trials. An alternative possibility is that the decision threshold changes across trials (Pedersen, Frank, & Biele, 2017). For example, if decision makers gradually "let their guard down," becoming less cautious over time as they become more familiar with the task, then they might require more evidence to make a decision in earlier trials (i.e., higher threshold) and less evidence in later trials (i.e., lower threshold). The expected result would be a decrease in response times (RT) across trials. In Experiment 2, we tested a second set of models with trial-dependent (decreasing) decision thresholds to determine whether they could better capture the aggregate RT patterns. In these models the decision threshold changes across trials according to the following functional form:

$$b_t = A + \frac{b_{sep,0}}{1 + \delta \cdot (t - 1)}$$

where b_t is the decision threshold on trial t, A is the upper bound of the start point distribution, $b_{sep,0}$ is the baseline (initial) decision threshold, and δ is a decay parameter. Note that if $\delta = 0$, the model reduces to a static threshold model: $b_t = A + b_{sep,0}$ for all t. When $\delta > 0$, the threshold will decrease across trials toward the start point upper bound. Higher values of the decay parameter lead to steeper decreases in the decision threshold across trials. When fitting this model to individual choice-RT data in Experiment 2, we used the following priors:

$$b_{sep,0} \sim Gamma(6, 100)$$

$$\delta \sim Gamma(1.01, 0.1)$$

(both using the shape-scale parameterization). As shown in S7 Fig, the models with trial-dependent decision thresholds fit the aggregate RT curves better than the models with static thresholds. The mean parameter estimates were $b_{sep,0} = 310.69$ (SD = 165.80), $\delta = 0.06$ (SD = 0.11), and $A = 299.14$ (SD = 140.38) for the "softmax(Q + gaze)" model. The mean decay parameter δ was significantly above zero, $t(49) = 3.78$, $p < .001$.

Pedersen ML, Frank MJ, Biele G. The drift diffusion model as the choice rule in reinforcement learning. *Psychon Bull Rev*. 2017;24(4):1234-51.
