# Supplementary material for: A reinforcement learning and sequential sampling model constrained by gaze data
Source: PLoS Comput Biol. 2026 Mar 6;22(3):e1014052. doi: 10.1371/journal.pcbi.1014052 (PMC12991361; doi:10.1371/journal.pcbi.1014052)
Supplement: S3 Appendix — (PDF) [file pcbi.1014052.s003.pdf]

S3 Appendix: Model-based analysis of individual differences

Different participants were best described by different models. To characterize some of these individual differences, we used the estimated individual-level coefficients from mixed effects regressions, which represent person-specific effects of value and gaze on choice accuracy and response time, to predict one model's relative advantage over another for individual participants. For this analysis, we use the difference in accumulative one-step-ahead prediction error (ΔAPE) between a particular model and Model 7 ("softmax(Q) + gaze") as the dependent variable, where positive numbers indicate an advantage for Model 7, and negative numbers indicate an advantage for the other model. We only analyzed the data from Experiment 1 to avoid the distinction between absolute and relative values.

First, given that the "Q + gaze" model (Model 3) predicts a negative effect of *overall value* on RT, while Model 7 predicts a negative effect of the *value difference* (S1 Fig), we expected that the individual-level coefficients for these two effects (derived from the model in S10 Table) would be predictive of Model 7's advantage over Model 3, but in opposite directions. Indeed, the slope for the effect of EV difference was negative (-88.51) and the slope for the effect of overall EV was positive (46.71) with both ps < .001 (multiple regression; adjusted $R^2 = .22$); thus, Model 7 was better at accounting for participants who exhibited a more negative effect of EV difference and a less negative effect of summed EV on log RT.

Second, given that the "Q * gaze" model (Model 2) predicts a stronger effect of gaze on choice for options with higher overall value, while Model 7 predicts a constant gaze effect (S2 Fig), we expected that individual-level coefficients for the interaction between proportional gaze difference and overall EV would be negatively associated with the advantage of Model 7 over Model 2. We first fit a generalized linear mixed-effects model predicting the probability of choosing the correct symbol on each learning phase trial as a function of the overall (summed) EV of the available options, the proportional gaze difference for the available options (correct minus incorrect), and the interaction. Although the fixed group-level interaction was not significant (p = .97), the correlation between the individual-level interaction effects and the advantage of Model 7 over Model 2 was negative and significant (r = -0.34, p = .002). Thus, Model 7 did not perform as well for participants in the first experiment who exhibited a stronger gaze effect for higher-value choice pairs.
