# Supplementary figures and images for: A reinforcement learning and sequential sampling model constrained by gaze data
Source: PLoS Comput Biol. 2026 Mar 6;22(3):e1014052. doi: 10.1371/journal.pcbi.1014052 (PMC12991361; doi:10.1371/journal.pcbi.1014052)

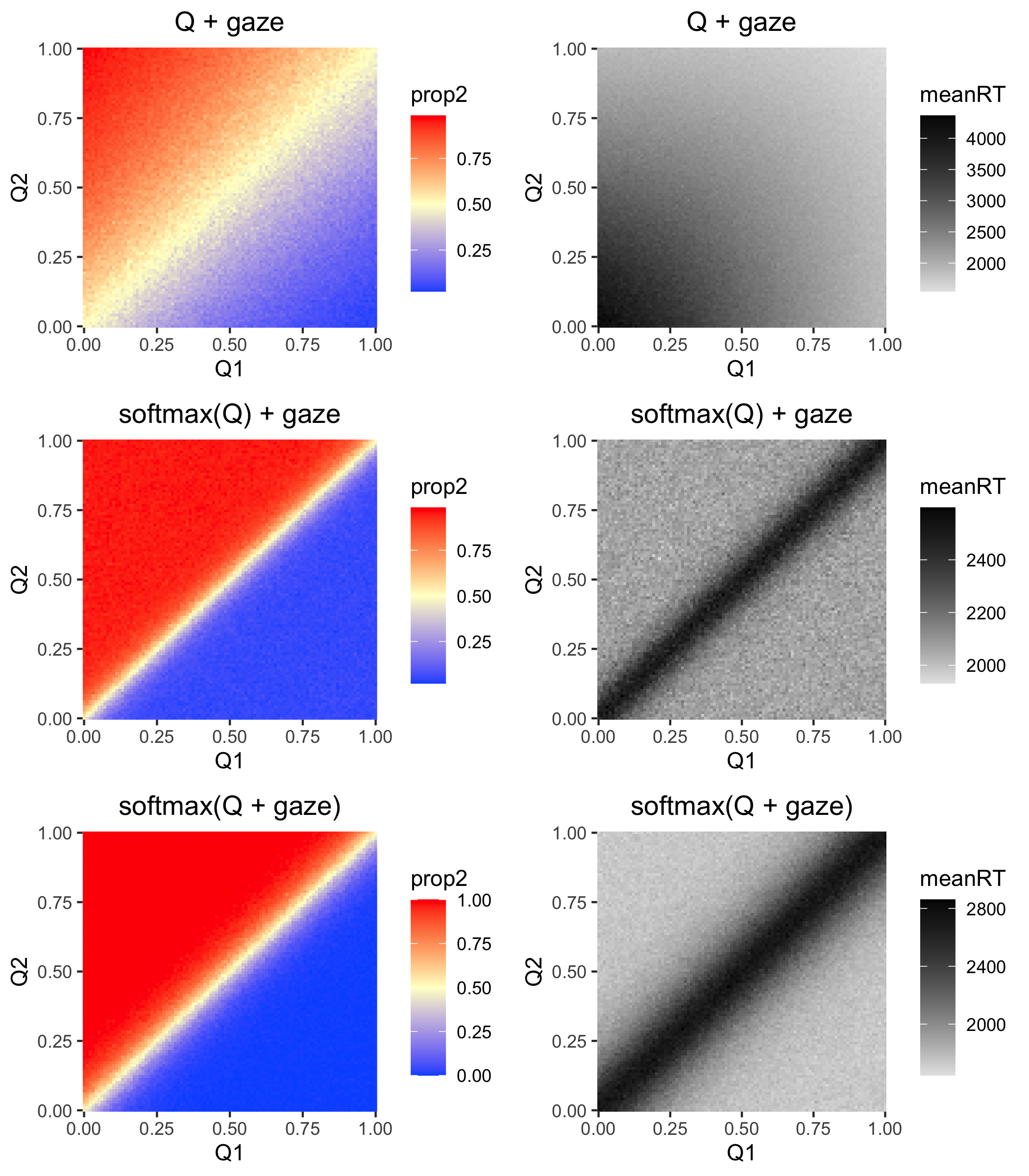

Supplement: S1 Fig — Each model was simulated 500 times for each combination of Q-values (proportional gaze was set to 0.5 for both alternatives). The left panels show the proportion of times the second alternative was chosen (prop2) and the right panels show the mean RT (ms) across the 500 runs for each Q-value combination. Models were simulated using the mean parameter estimates from Experiment 1 (Q + gaze: βQ=0.32 , βgaze=0.27 , A=631.09 , b=1086.19 , t0=133.44 ; softmax(Q) + gaze: βQ=0.28 , βgaze=0.27 , θ=18.35 , A=615.96 , b=1069.49 , t0=135.13 ; softmax(Q + gaze): βQ=0.47 , βgaze=0.28 , θ=8.82 , A=562.17 , b=993.64 , t0=139.61 ). The Q + gaze model exhibits a noisier decision boundary and produces mean RTs that depend on the magnitude of the Q-values, with faster RTs for larger magnitudes. The softmax models exhibit a sharper decision boundary and mean RTs that depend only on the difference between Q values, with faster RTs for larger differences. (PNG) [file pcbi.1014052.s004.png]

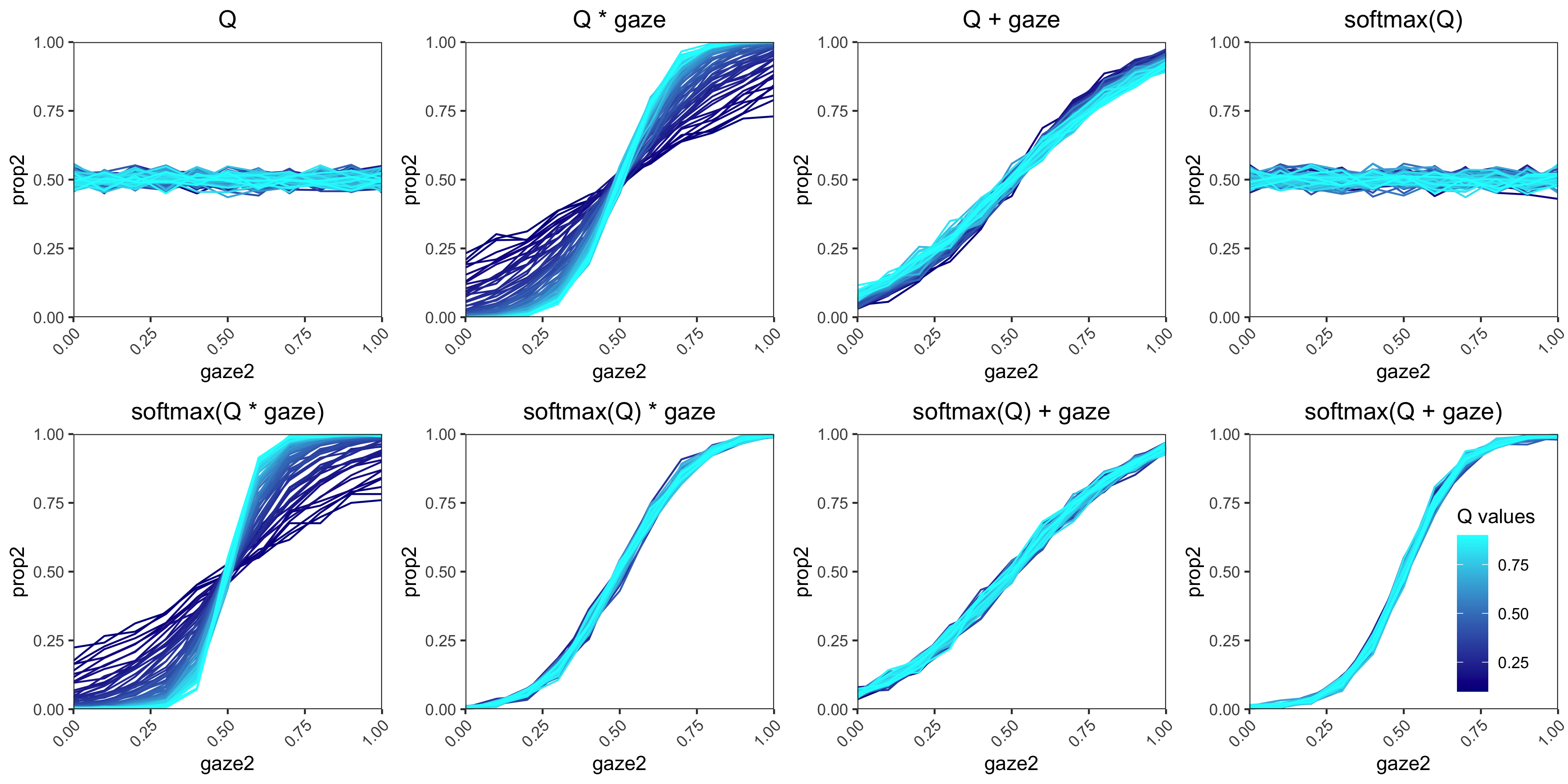

Supplement: S2 Fig — Models were simulated 500 times for each combination of Q-value magnitude, ranging from 0.1 to 0.9 in increments of 0.01, and proportional gaze allocated to the second alternative (gaze2), ranging from 0 to 1 in increments of 0.1. Both options were given the same Q-value in the simulations (i.e., one of the magnitudes listed above). The y-axis in each panel is the proportion of times the second alternative was chosen (prop2). The models without gaze data show no gaze effects. The Q * gaze and softmax(Q * gaze) models exhibit stronger gaze effects when the options have larger Q-values. The Q + gaze model, in contrast, exhibits a slightly stronger gaze effect when the Q-values are smaller. Models were simulated using the mean parameter estimates from Experiment 1. (PNG) [file pcbi.1014052.s005.png]

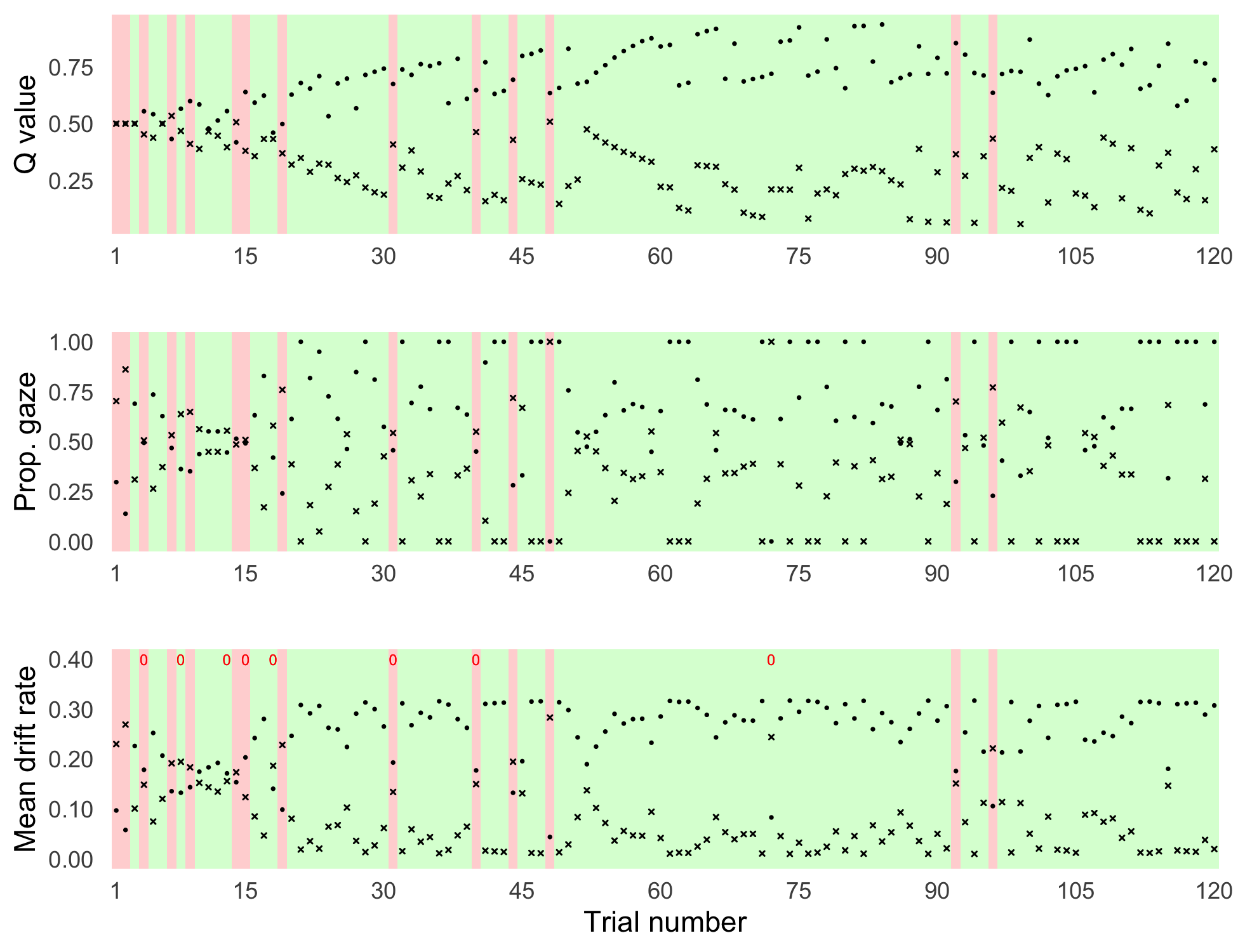

Supplement: S3 Fig — Trial-to-trial Q-values, proportional gaze data, and mean drift rates for participant 32 in the learning phase of Experiment 2 (softmax(Q + gaze) model parameter estimates: α=.15 , wrel=.66 , βQ=0.33 , βgaze=1.03 , θ=2.06 , A=151.31 , b=281.12 , t0=77.71 ). The background colors indicate the participant’s actual choice (green = correct option, red = incorrect option). The black circles represent the correct option on each trial; the black x’s represent the incorrect option. The model correctly predicted 112 out of 120 (93%) of this participant’s choices (in the mean drift rate plot, the red 0’s indicate inaccurate predictions). Because the learning contexts were randomly ordered, the correct and incorrect options do not refer to the same underlying symbols on every trial. (PNG) [file pcbi.1014052.s006.png]

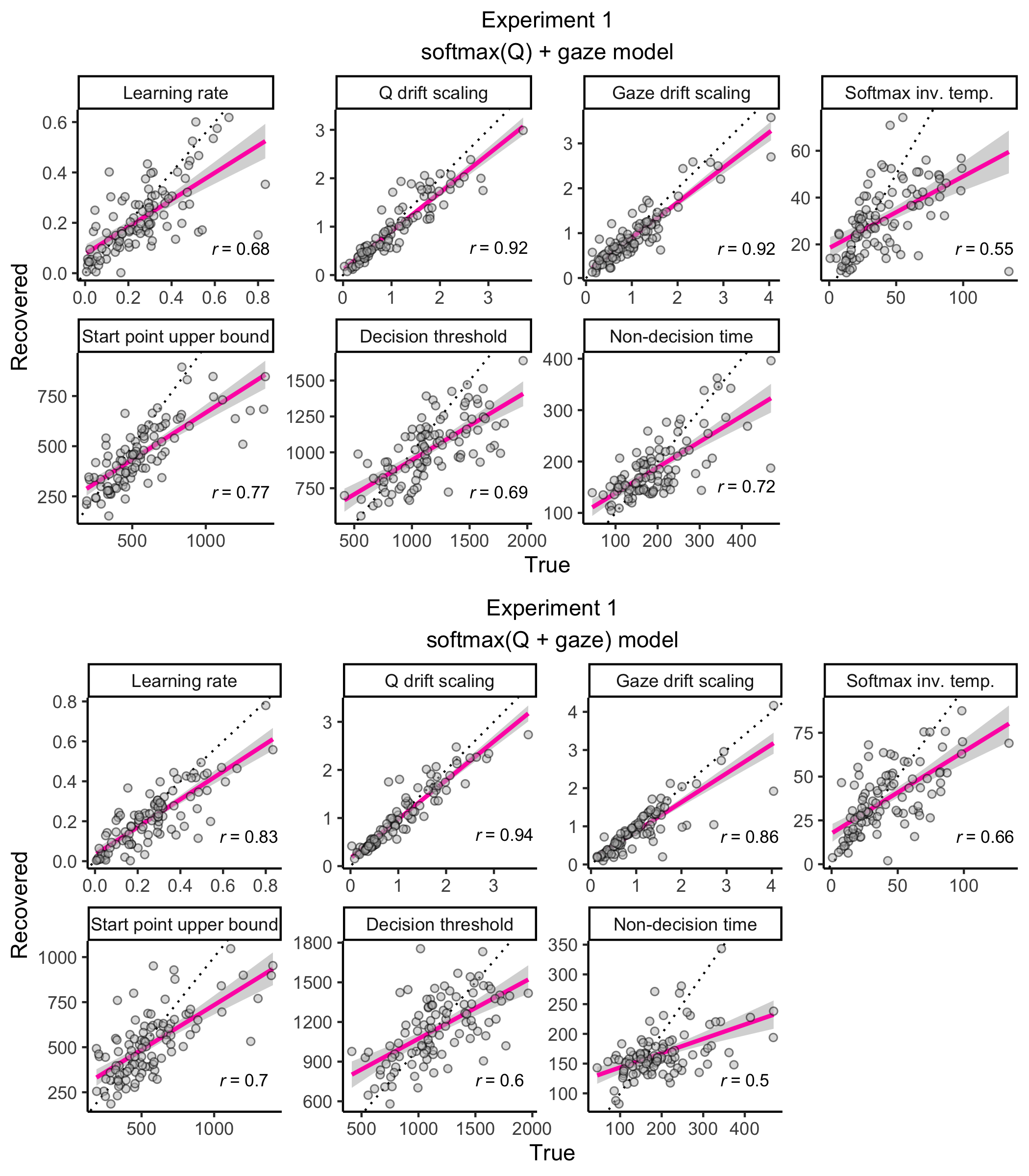

Supplement: S4 Fig — The “softmax(Q) + gaze” and “softmax(Q + gaze)” models were simulated 100 times in the task from the first experiment (60 trials) using parameter values drawn from the prior distributions (see main text, Materials and methods). Then, the models were fit to the 100 simulated data sets to assess their ability to recover the true, data-generating parameters. Relationships between the generating and recovered parameters are shown with regression lines overlaid. The “softmax(Q) + gaze” model had the lowest accumulative one-step-ahead prediction error in Experiment 1, averaged across participants. (PNG) [file pcbi.1014052.s007.png]

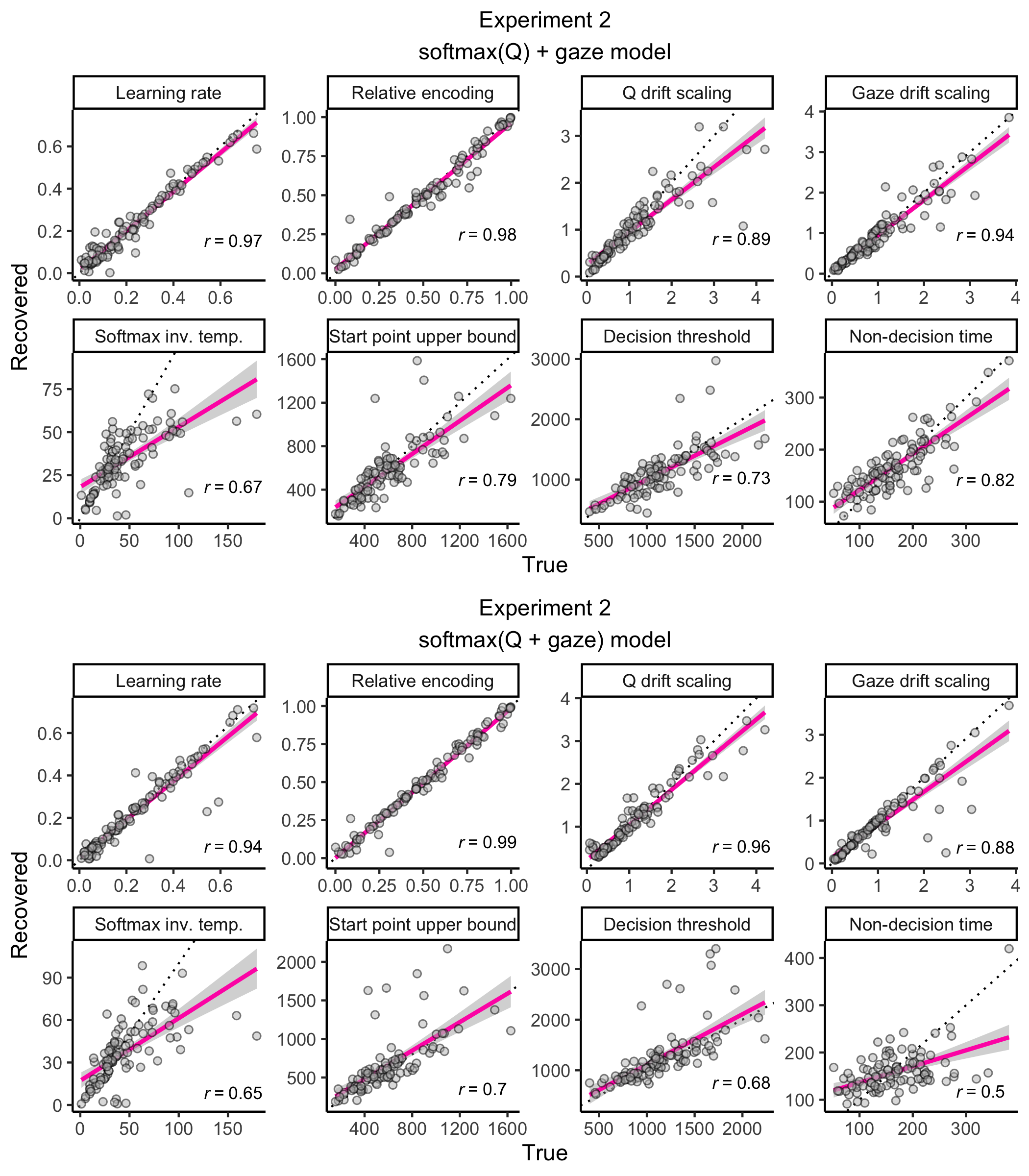

Supplement: S5 Fig — The “softmax(Q) + gaze” and “softmax(Q + gaze)” models were simulated 100 times in the task from the second experiment (232 trials) using parameter values drawn from the prior distributions (see main text, Materials and methods). Then, the models were fit to the 100 simulated data sets to assess their ability to recover the true, data-generating parameters. Relationships between the generating and recovered parameters are shown with regression lines overlaid. The “softmax(Q + gaze)” model had the lowest accumulative one-step-ahead prediction error in Experiment 2, averaged across participants. (PNG) [file pcbi.1014052.s008.png]

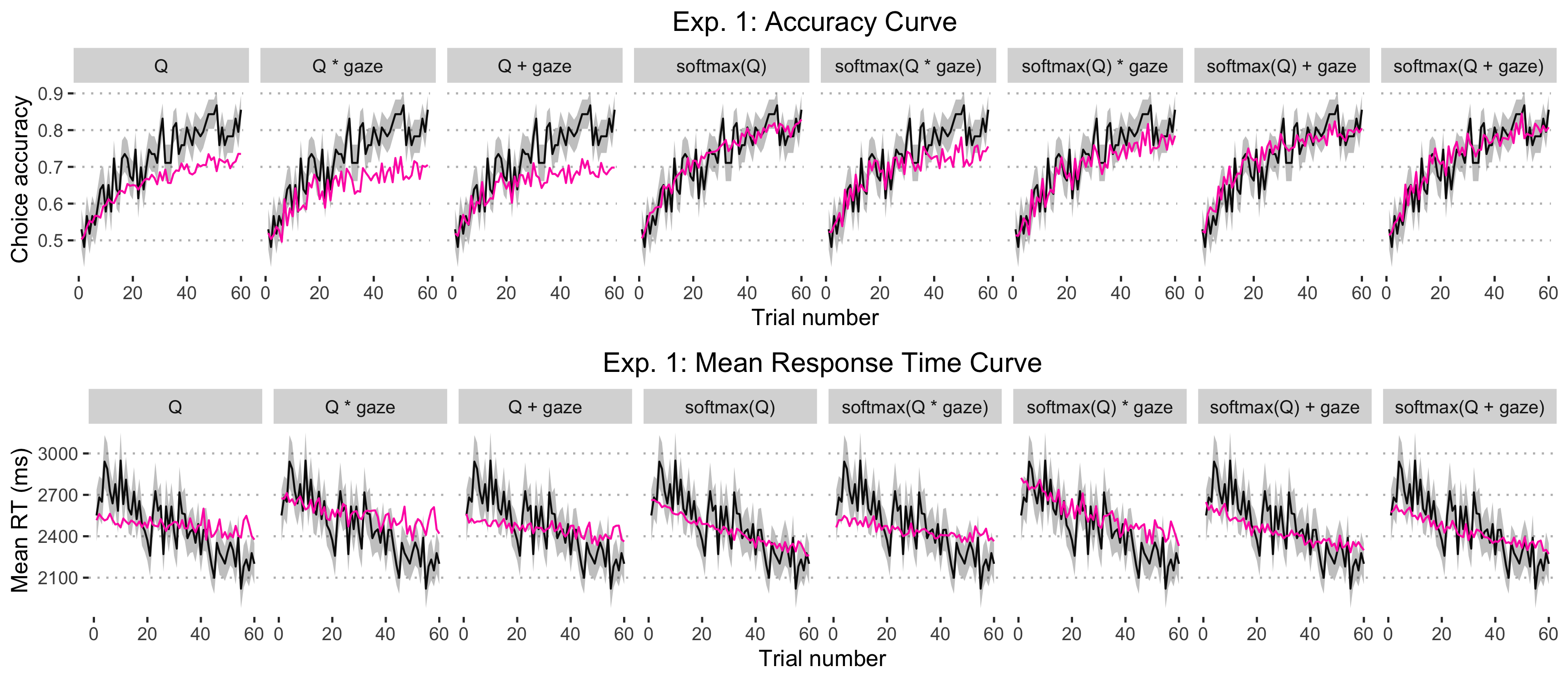

Supplement: S6 Fig — Proportion of correct choices and mean RT across learning trials. Error ribbons represent ±1 standard error. (PNG) [file pcbi.1014052.s009.png]

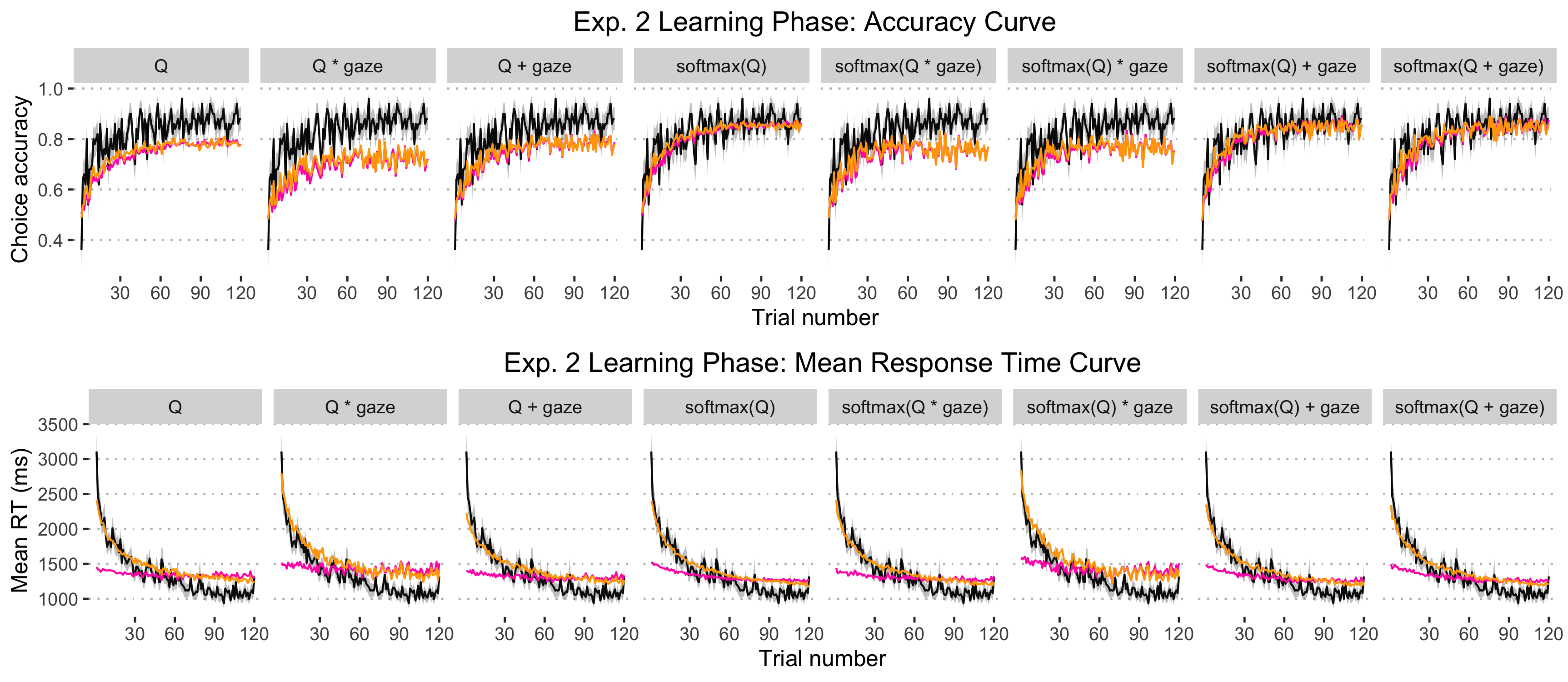

Supplement: S7 Fig — Proportion of correct choices and mean RT across learning trials. Error ribbons represent ±1 standard error. The purple lines show the fit of the original model with a static decision threshold. The orange lines show the fit of the modified model with a trial-dependent (decreasing) decision threshold, which was better at capturing the steep, nonlinear decrease in mean RT across the learning phase. (PNG) [file pcbi.1014052.s010.png]

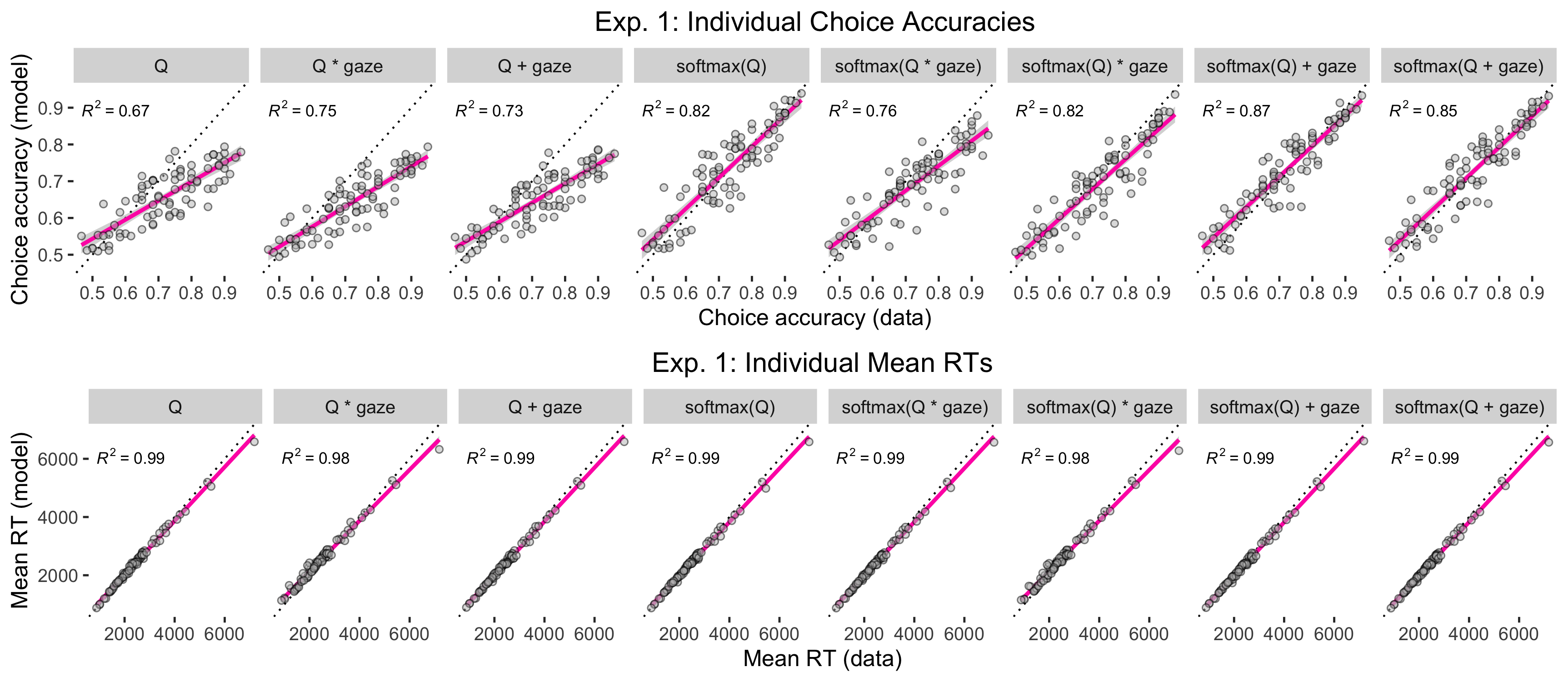

Supplement: S8 Fig — (PNG) [file pcbi.1014052.s011.png]

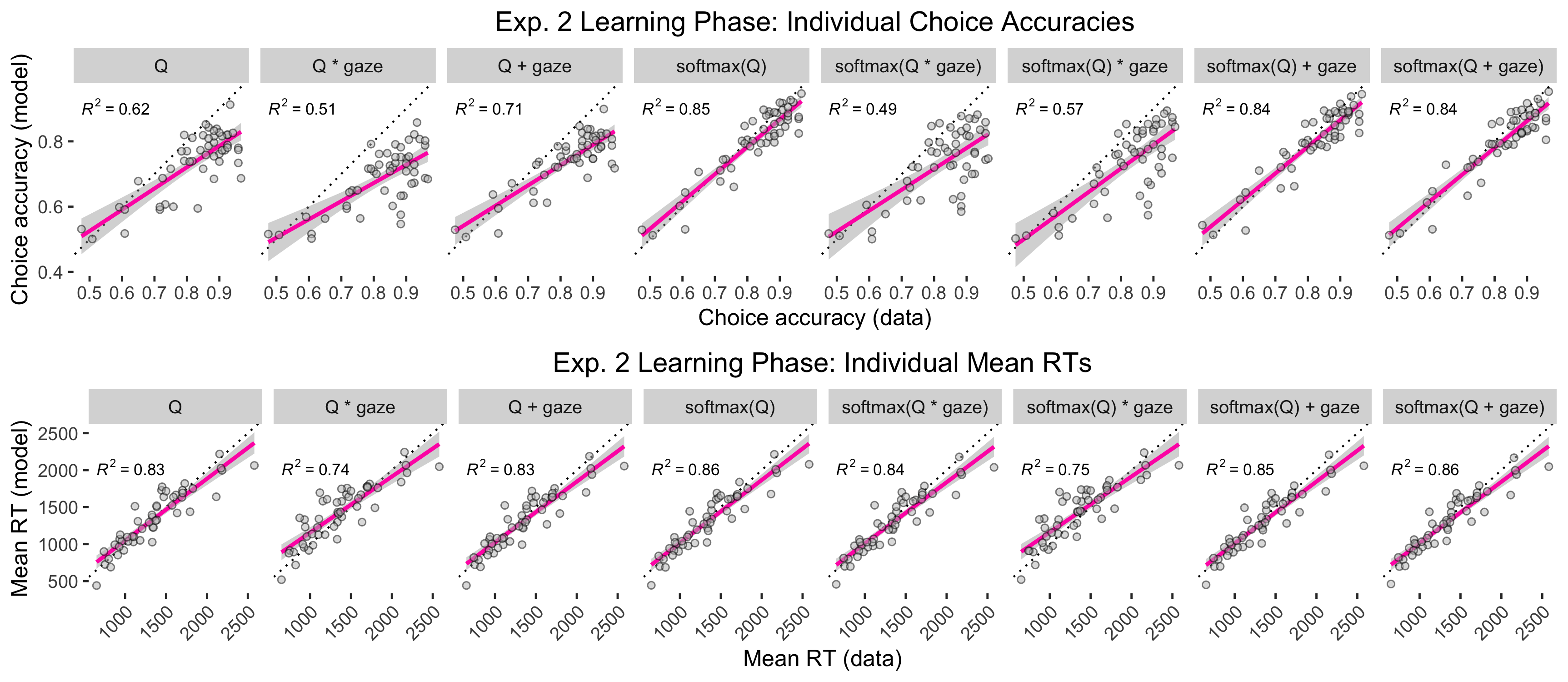

Supplement: S9 Fig — (PNG) [file pcbi.1014052.s012.png]

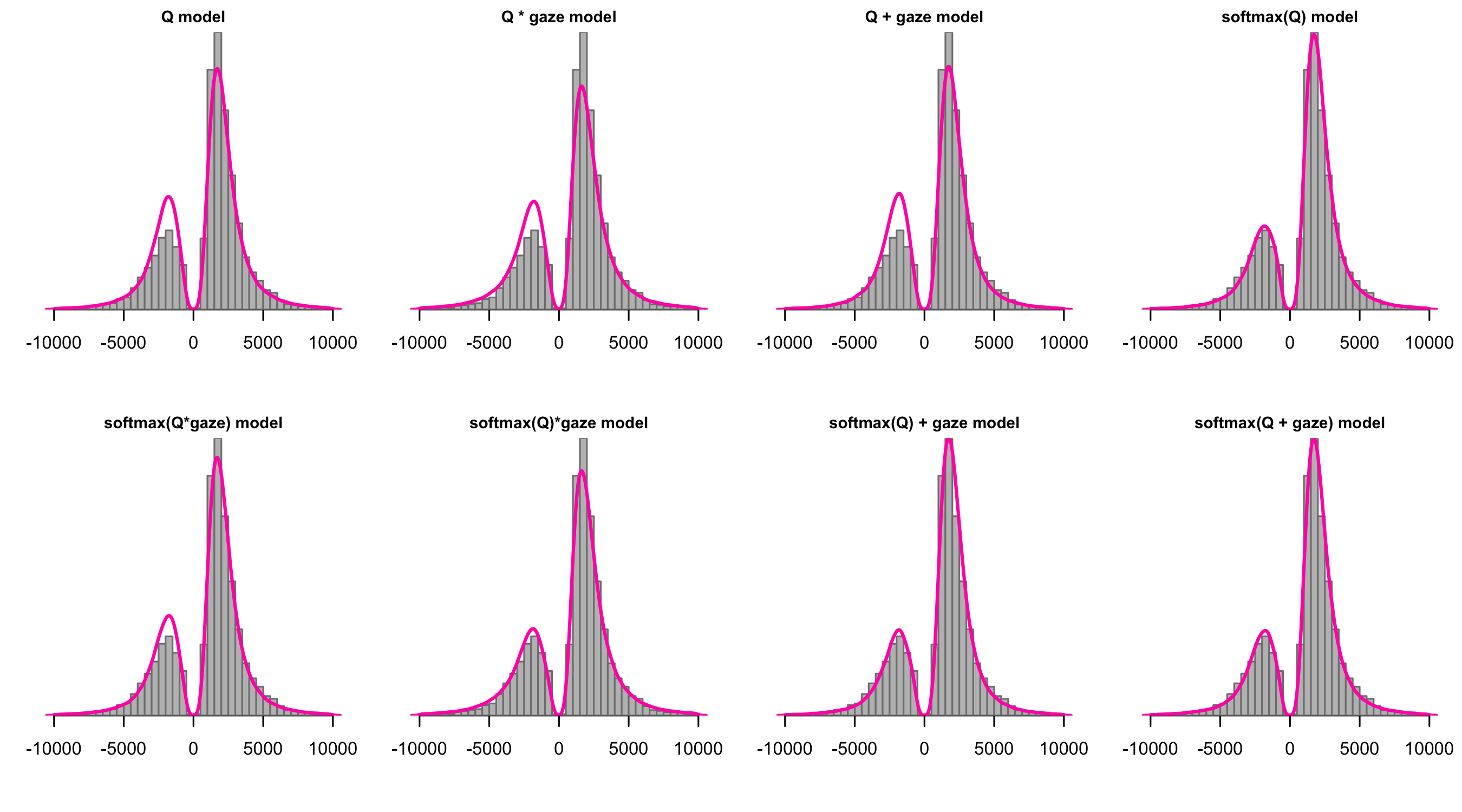

Supplement: S10 Fig — RT distributions for correct (maximizing) and incorrect (nonmaximizing) choices were pooled across participants. Incorrect RTs are negative for visualization purposes. (PNG) [file pcbi.1014052.s013.png]

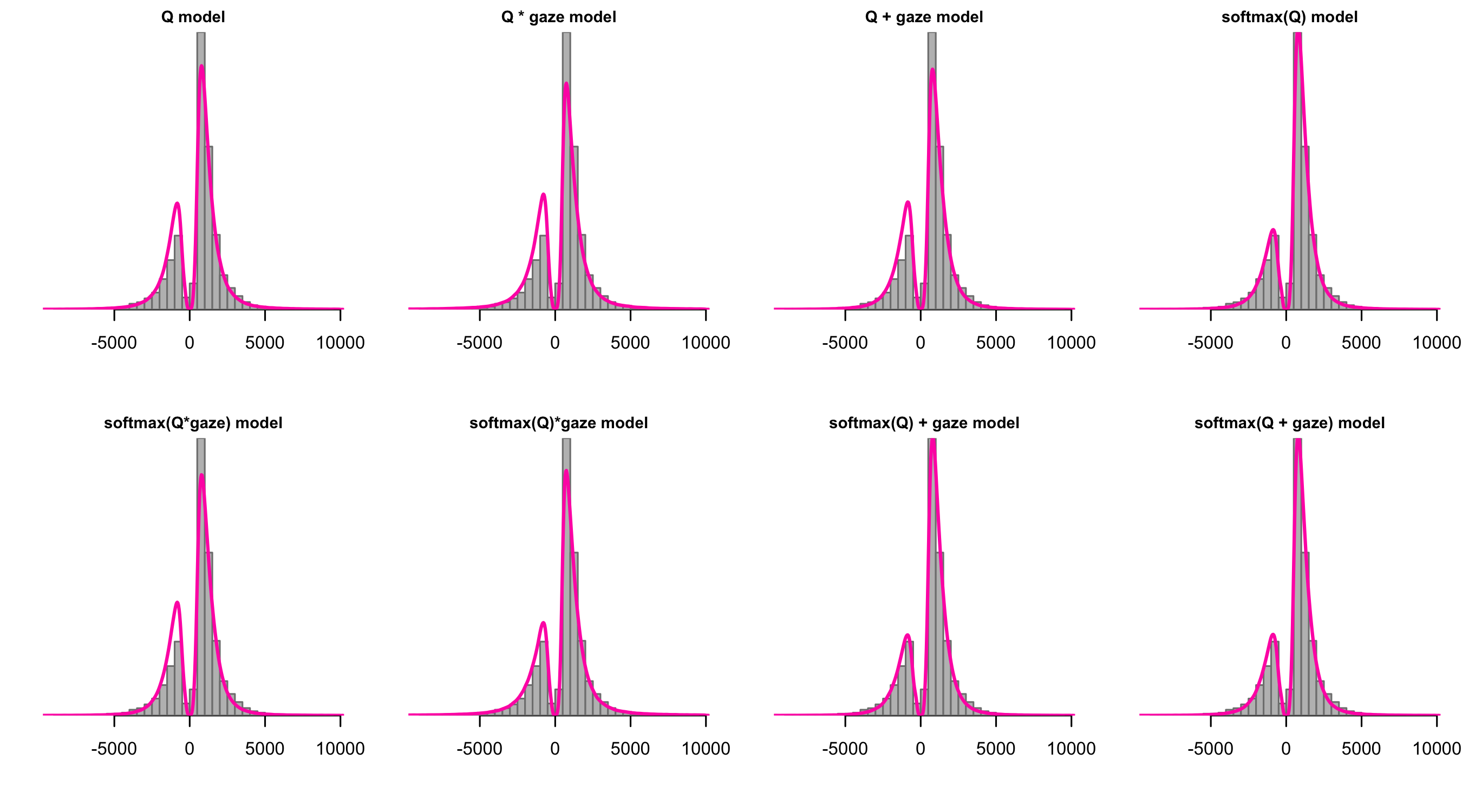

Supplement: S11 Fig — RT distributions for correct (maximizing) and incorrect (nonmaximizing) choices were pooled across participants. Incorrect RTs are negative for visualization purposes. (PNG) [file pcbi.1014052.s014.png]

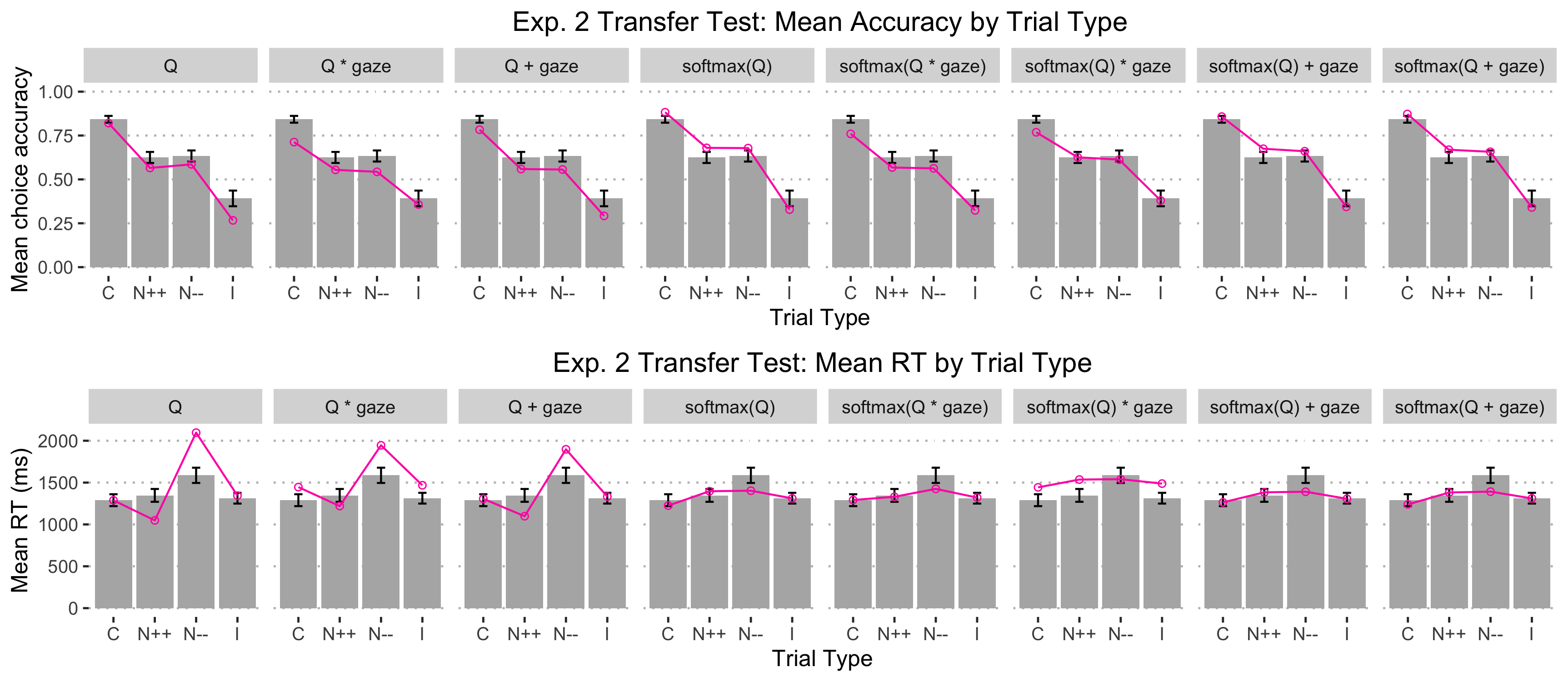

Supplement: S12 Fig — Mean choice accuracy and mean RT as a function of trial type (C = congruent, N++ = neutral with both options having high relative values, N-- = neutral with both options having low relative values, I = incongruent). Error bars represent ±1 standard error. (PNG) [file pcbi.1014052.s015.png]

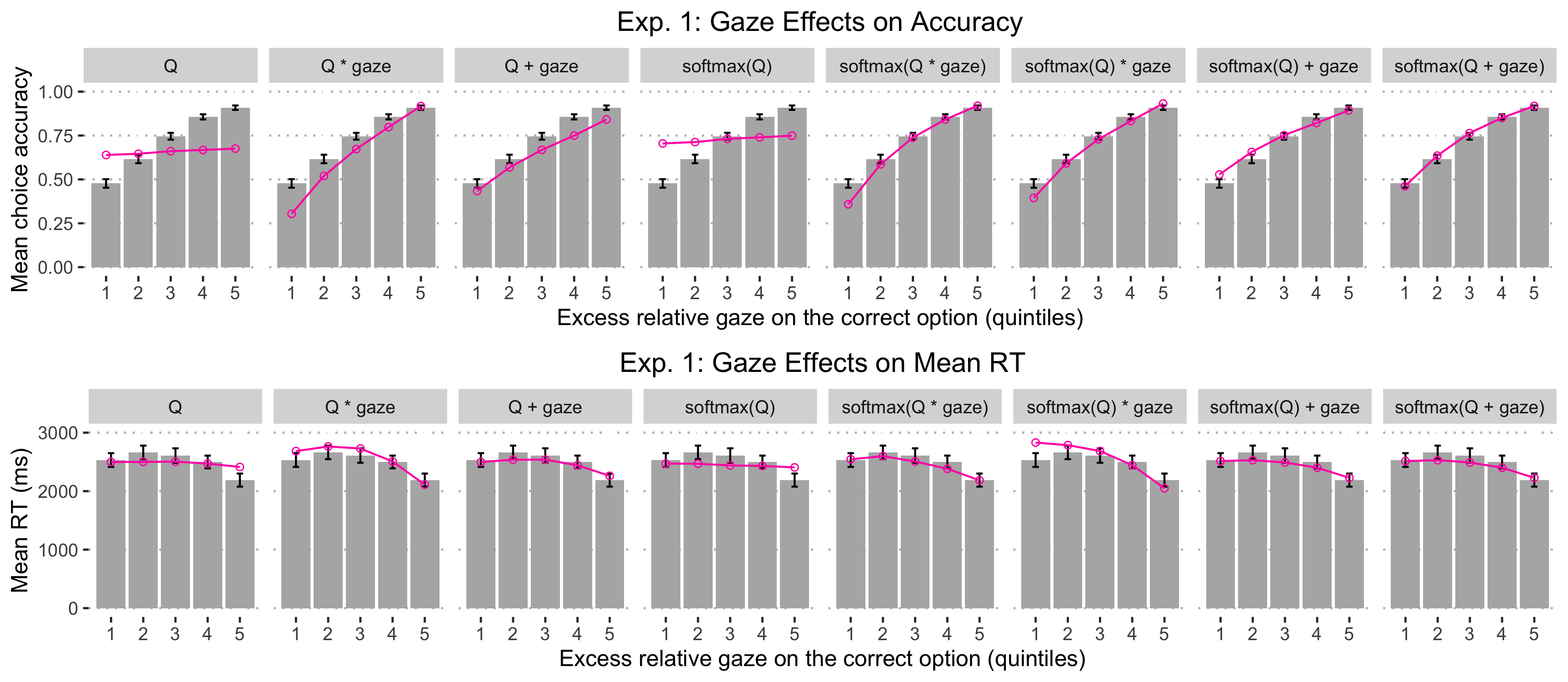

Supplement: S13 Fig — Mean choice accuracy (top) and mean RT (bottom) as a function of the excess proportional gaze on the correct option, broken up into five quintiles. The quintiles were constructed by taking the difference between the proportional gaze for the correct (higher valued) and incorrect (lower valued) symbols on each trial, sorting the difference scores, and dividing them into five equal-sized bins, separately for each participant. The higher the quintile, the longer the correct option was fixated relative to the incorrect option. Error bars represent ±1 standard error. (PNG) [file pcbi.1014052.s016.png]

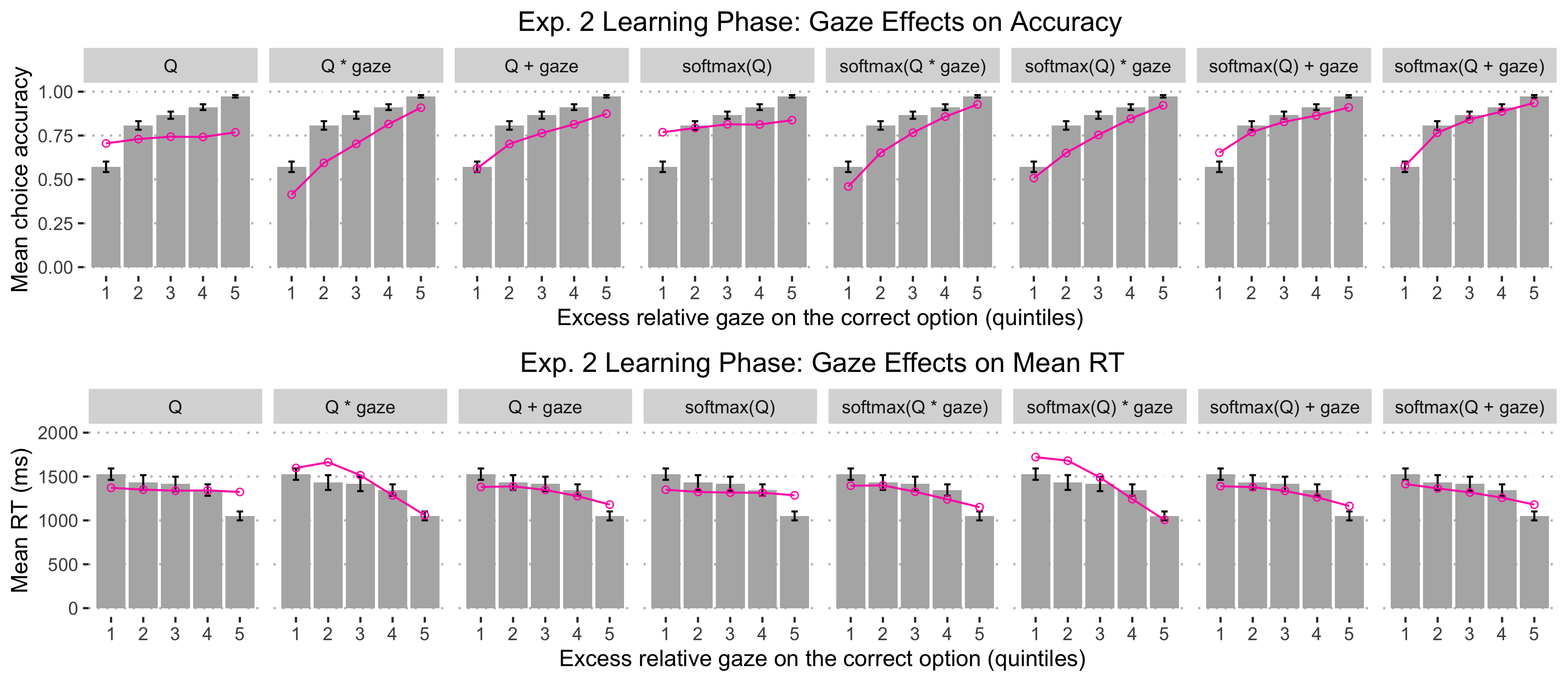

Supplement: S14 Fig — Mean choice accuracy (top) and mean RT (bottom) as a function of the excess proportional gaze on the correct option, broken up into five quintiles. The quintiles were constructed by taking the difference between the proportional gaze for the correct (higher valued) and incorrect (lower valued) symbols on each trial, sorting the difference scores, and dividing them into five equal-sized bins, separately for each participant. The higher the quintile, the longer the correct option was fixated relative to the incorrect option. Error bars represent ±1 standard error. (PNG) [file pcbi.1014052.s017.png]

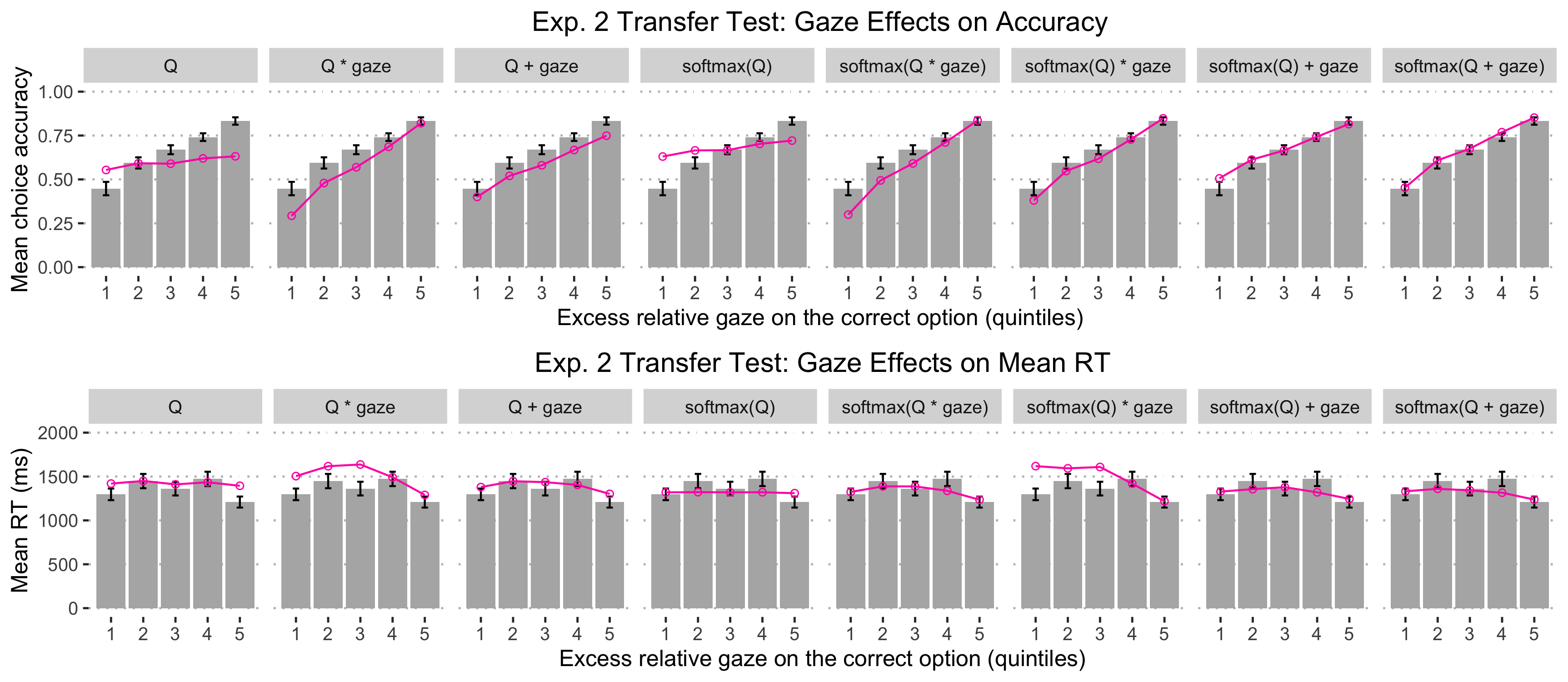

Supplement: S15 Fig — Mean choice accuracy (top) and mean RT (bottom) as a function of the excess proportional gaze on the correct option, broken up into five quintiles. The quintiles were constructed by taking the difference between the proportional gaze for the correct (higher valued) and incorrect (lower valued) symbols on each trial, sorting the difference scores, and dividing them into five equal-sized bins, separately for each participant. The higher the quintile, the longer the correct option was fixated relative to the incorrect option. Error bars represent ±1 standard error. (PNG) [file pcbi.1014052.s018.png]
