# Supplementary material for: A reinforcement learning and sequential sampling model constrained by gaze data
Source: PLoS Comput Biol. 2026 Mar 6;22(3):e1014052. doi: 10.1371/journal.pcbi.1014052 (PMC12991361; doi:10.1371/journal.pcbi.1014052)
Supplement: S1 Table — (PDF) [file pcbi.1014052.s019.pdf]

S1 Table: Multiple Regression Predicting Individual Choice Accuracy from RL-SSM Parameters (Experiment 1)

**Predictor**	b	SE	t	p
Intercept	0.50	0.12	4.17	< .001
Learning rate (α)	1.01	0.26	3.97	< .001
Q drift scaling (β_Q)	0.28	0.10	2.78	.007
Gaze drift scaling (β_{gaze})	-0.21	0.098	-2.10	.039
Softmax inverse temperature (θ)	0.0093	0.0017	5.47	< .001
Start point upper bound (A)	-0.00011	9.12e-5	-1.20	.23
Decision threshold (b)	0.00015	5.98e-5	2.52	.014
Non-decision time (t_0)	-0.00077	0.00079	-0.98	.33

Note. Parameters estimated from the winning model in Experiment 1, "softmax(Q) + gaze." Adjusted $R^2 = .36$, $F(7, 75) = 7.57$, $p < .001$.
