# Supplementary material for: A reinforcement learning and sequential sampling model constrained by gaze data
Source: PLoS Comput Biol. 2026 Mar 6;22(3):e1014052. doi: 10.1371/journal.pcbi.1014052 (PMC12991361; doi:10.1371/journal.pcbi.1014052)
Supplement: S2 Table — (PDF) [file pcbi.1014052.s020.pdf]

S2 Table: Multiple Regression Predicting Individual Choice Accuracy from RL-SSM Parameters (Experiment 2: Learning Phase)

**Predictor**	b	SE	t	p
Intercept	0.73	0.11	6.93	< .001
Learning rate (α)	0.29	0.09	3.27	.002
Relative encoding (w_{rel})	0.10	0.04	2.46	.018
Q drift scaling (β_Q)	0.11	0.16	0.65	.52
Gaze drift scaling (β_{gaze})	-0.09	0.05	-1.56	.13
Softmax inverse temperature (θ)	0.0016	0.0019	0.85	.40
Start point upper bound (A)	-0.0014	0.00037	-3.68	< .001
Decision threshold (b)	0.00097	0.00032	2.98	.005
Non-decision time (t_0)	-0.00053	0.00055	-0.97	.34

Note. Parameters estimated from the winning model in Experiment 2, “softmax(Q + gaze).” Adjusted $R^2 = .41$, $F(8, 41) = 5.29$, $p < .001$.
