# Supplementary material for: A reinforcement learning and sequential sampling model constrained by gaze data
Source: PLoS Comput Biol. 2026 Mar 6;22(3):e1014052. doi: 10.1371/journal.pcbi.1014052 (PMC12991361; doi:10.1371/journal.pcbi.1014052)
Supplement: S3 Table — (PDF) [file pcbi.1014052.s021.pdf]

S3 Table: Multiple Regression Predicting Individual Mean RT from RL-SSM Parameters (Experiment 1)

**Predictor**	b	SE	t	p
Intercept	1857.03	336.31	5.52	< .001
Learning rate (α)	-161.66	721.09	-0.22	.82
Q drift scaling (β_Q)	-3582.36	286.16	-12.52	< .001
Gaze drift scaling (β_{gaze})	-3377.83	277.07	-12.19	< .001
Softmax inverse temperature (θ)	-4.48	4.79	-0.94	.35
Start point upper bound (A)	-1.74	0.26	-6.78	< .001
Decision threshold (b)	3.32	0.17	19.62	< .001
Non-decision time (t_0)	1.35	2.22	0.61	.55

Note. Parameters estimated from the winning model in Experiment 1, “softmax(Q) + gaze.” Adjusted $R^2 = .92$, $F(7, 75) = 136.5$, $p < .001$.
