# Supplementary material for: A reinforcement learning and sequential sampling model constrained by gaze data
Source: PLoS Comput Biol. 2026 Mar 6;22(3):e1014052. doi: 10.1371/journal.pcbi.1014052 (PMC12991361; doi:10.1371/journal.pcbi.1014052)
Supplement: S4 Table — (PDF) [file pcbi.1014052.s022.pdf]

S4 Table: Multiple Regression Predicting Individual Mean RT from RL-SSM Parameters (Experiment 2: Learning Phase)

**Predictor**	b	SE	t	p
Intercept	949.50	220.50	4.31	< .001
Learning rate (α)	-192.70	185.50	-1.04	.31
Relative encoding (w_{rel})	3.06	88.22	0.04	.97
Q drift scaling (β_Q)	-3009.00	337.80	-8.91	< .001
Gaze drift scaling (β_{gaze})	172.40	114.30	1.51	.14
Softmax inverse temperature (θ)	-0.077	3.90	-0.02	.98
Start point upper bound (A)	-0.64	0.77	-0.82	.42
Decision threshold (b)	2.85	0.67	4.23	< .001
Non-decision time (t_0)	3.20	1.14	2.80	.008

Note. Parameters estimated from the winning model in Experiment 2, “softmax(Q + gaze).” Adjusted $R^2 = .81$, $F(8, 41) = 27.09$, $p < .001$.
