# Supplementary material for: A reinforcement learning and sequential sampling model constrained by gaze data
Source: PLoS Comput Biol. 2026 Mar 6;22(3):e1014052. doi: 10.1371/journal.pcbi.1014052 (PMC12991361; doi:10.1371/journal.pcbi.1014052)
Supplement: S5 Table — (PDF) [file pcbi.1014052.s023.pdf]

S5 Table: Multiple Regression Predicting Incongruent Trial Accuracy from RL-SSM Parameters (Experiment 2: Transfer Test)

**Predictor**	b	SE	t	p
Intercept	0.75	0.19	3.91	< .001
Learning rate (α)	0.30	0.16	1.87	.068
Relative encoding (w_{rel})	-0.72	0.076	-9.42	< .001
Q drift scaling (β_Q)	-0.035	0.29	-0.12	.91
Gaze drift scaling (β_{gaze})	0.043	0.099	0.43	.67
Softmax inverse temperature (θ)	0.0054	0.0034	1.59	.12
Start point upper bound (A)	-0.00049	0.00067	-0.73	.47
Decision threshold (b)	0.00034	0.00058	0.58	.57
Non-decision time (t_0)	-0.00039	0.00099	-0.39	.70

Note. Parameters estimated from the winning model in Experiment 2, "softmax(Q + gaze)." Adjusted $R^2 = .74$, $F(8, 41) = 18.39$, $p < .001$.
