# Supplementary material for: A reinforcement learning and sequential sampling model constrained by gaze data
Source: PLoS Comput Biol. 2026 Mar 6;22(3):e1014052. doi: 10.1371/journal.pcbi.1014052 (PMC12991361; doi:10.1371/journal.pcbi.1014052)
Supplement: S6 Table — (PDF) [file pcbi.1014052.s024.pdf]

S6 Table: Linear Mixed-Effects Model Predicting Proportional Gaze Advantage for the Correct Option from Trial Number, EV Difference, and Overall Expected Value in Experiment 1

**Fixed Effects**	b	SE	t	p
Intercept	0.059	0.0062	9.44	< .001
EV Difference	0.035	0.0052	6.60	< .001
Trial Number	0.018	0.0052	3.44	< .001
Overall EV	0.013	0.0070	1.89	0.062
Trial Number × EV Difference	-0.0071	0.0051	-1.37	0.17
Trial Number × Overall EV	0.0080	0.0050	1.60	0.11
**Random Effects**	Variance			
Intercept	0.0014			
EV Difference	0.00043			
Trial Number	0.00035			
Overall EV	0.0022			
Trial Number × EV Difference	0.00031			
Trial Number × Overall EV	0.00016			
Residual	0.11			

Note. Improvement over intercept-only model: $\chi^2(25) = 83.61$, $p < .001$
