# Supplementary material for: A reinforcement learning and sequential sampling model constrained by gaze data
Source: PLoS Comput Biol. 2026 Mar 6;22(3):e1014052. doi: 10.1371/journal.pcbi.1014052 (PMC12991361; doi:10.1371/journal.pcbi.1014052)
Supplement: S7 Table — (PDF) [file pcbi.1014052.s025.pdf]

S7 Table: Linear Mixed-Effects Model Predicting Proportional Gaze Advantage for the Correct Option from Trial Number and Overall Expected Value in the Learning Phase of Experiment 2

**Fixed Effects**	b	SE	t	p
Intercept	0.20	0.015	12.65	< .001
Trial Number	0.058	0.0093	6.20	< .001
Overall EV	0.0026	0.0083	0.31	0.76
Trial Number × Overall EV	-0.0067	0.0084	-0.79	0.43
**Random Effects**	Variance			
Intercept	0.010			
Trial Number	0.0023			
Overall EV	0.0015			
Trial Number × Overall EV	0.0015			
Residual	0.24			

Note. Improvement over intercept-only model: $\chi^2(12) = 122.8$, $p < .001$
