# Supplementary material for: A reinforcement learning and sequential sampling model constrained by gaze data
Source: PLoS Comput Biol. 2026 Mar 6;22(3):e1014052. doi: 10.1371/journal.pcbi.1014052 (PMC12991361; doi:10.1371/journal.pcbi.1014052)
Supplement: S8 Table — (PDF) [file pcbi.1014052.s026.pdf]

S8 Table: Logistic Mixed-Effects Model Predicting Choice Accuracy from Trial Number, EV Difference, Overall EV, and Proportional Gaze Advantage for the Correct Option in Experiment 1

**Fixed Effects**	b	SE	z	p
Intercept	1.63	0.13	12.28	< .001
Trial Number	0.71	0.077	9.19	< .001
EV Difference	0.67	0.072	9.20	< .001
Overall EV	-0.0091	0.052	-0.17	0.862
Gaze Difference	1.076	0.074	14.58	< .001
Trial Number × EV Difference	0.32	0.067	4.77	< .001
Trial Number × Overall EV	0.019	0.052	0.37	0.71
Trial Number × Gaze Difference	-0.14	0.060	-2.29	0.022
**Random Effects**	Variance			
Intercept	1.13			
Trial Number	0.21			
EV Difference	0.17			
Overall EV	0.076			
Gaze Difference	0.21			
Trial Number × EV Difference	0.14			
Trial Number × Overall EV	0.072			
Trial Number × Gaze Difference	0.053			

Note. Improvement over no-gaze model: $\chi^2(17) = 708.15$, $p < .001$
