# Supplementary material for: A reinforcement learning and sequential sampling model constrained by gaze data
Source: PLoS Comput Biol. 2026 Mar 6;22(3):e1014052. doi: 10.1371/journal.pcbi.1014052 (PMC12991361; doi:10.1371/journal.pcbi.1014052)
Supplement: S9 Table — (PDF) [file pcbi.1014052.s027.pdf]

S9 Table: Logistic Mixed-Effects Model Predicting Choice Accuracy from Trial Number, Overall EV, and Proportional Gaze Advantage for the Correct Option in the Learning Phase of Experiment 2

**Fixed Effects**	b	SE	z	p
Intercept	2.59	0.19	13.54	< .001
Trial Number	0.64	0.11	5.80	< .001
Overall EV	-0.03	0.079	-0.38	0.71
Gaze Difference	1.33	0.097	13.66	< .001
Trial Number × Overall EV	0.030	0.061	0.49	0.63
Trial Number × Gaze Difference	-0.058	0.071	-0.82	0.41
**Random Effects**	Variance			
Intercept	1.49			
Trial Number	0.34			
Overall EV	0.13			
Gaze Difference	0.25			
Trial Number × Overall EV	0.033			
Trial Number × Gaze Difference	0.042			

Note. Improvement over no-gaze model: $\chi^2(13) = 852.3$, $p < .001$
