# Supplementary material for: A reinforcement learning and sequential sampling model constrained by gaze data
Source: PLoS Comput Biol. 2026 Mar 6;22(3):e1014052. doi: 10.1371/journal.pcbi.1014052 (PMC12991361; doi:10.1371/journal.pcbi.1014052)
Supplement: S10 Table — (PDF) [file pcbi.1014052.s028.pdf]

S10 Table: Linear Mixed-Effects Model Predicting log RT from Trial Number, EV Difference, Overall EV, and Proportional Gaze Advantage for the Correct Option in Experiment 1

**Fixed Effects**	b	SE	t	p
Intercept	7.68	0.042	180.75	< .001
Trial Number	-0.070	0.017	-4.11	< .001
EV Difference	-0.053	0.0067	-7.84	< .001
Overall EV	-0.036	0.0082	-4.43	< .001
Gaze Difference	-0.031	0.0068	-4.61	< .001
Trial Number × EV Difference	-0.0089	0.0053	-1.68	0.093
Trial Number × Overall EV	-0.019	0.0059	-3.16	0.0022
Trial Number × Gaze Difference	-0.0097	0.0052	-1.85	0.065
**Random Effects**	Variance			
Intercept	0.15			
Trial Number	0.022			
EV Difference	0.0015			
Overall EV	0.0034			
Gaze Difference	0.0015			
Trial Number × EV Difference	0			
Trial Number × Overall EV	0.00061			
Trial Number × Gaze Difference	0			
Residual	0.13			

Note. Improvement over no-gaze model: $\chi^2(4) = 31.45$, $p < .001$

Random effect correlations excluded from the model to aid convergence.
