# Supplementary material for: A reinforcement learning and sequential sampling model constrained by gaze data
Source: PLoS Comput Biol. 2026 Mar 6;22(3):e1014052. doi: 10.1371/journal.pcbi.1014052 (PMC12991361; doi:10.1371/journal.pcbi.1014052)
Supplement: S11 Table — (PDF) [file pcbi.1014052.s029.pdf]

S11 Table: Linear Mixed-Effects Model Predicting log RT from Trial Number, Overall EV, and Proportional Gaze Advantage for the Correct Option in the Learning Phase of Experiment 2

**Fixed Effects**	b	SE	t	p
Intercept	7.03	0.045	155.40	< .001
Trial Number	-0.19	0.015	-13.10	< .001
Overall EV	-0.0099	0.010	-0.99	0.33
Gaze Difference	-0.071	0.0091	-7.72	< .001
Trial Number × Overall EV	0.0031	0.0072	0.42	0.67
Trial Number × Gaze Difference	0.027	0.0080	3.32	.0018
**Random Effects**	Variance			
Intercept	0.101			
Trial Number	0.0091			
Overall EV	0.0034			
Gaze Difference	0.0025			
Trial Number × Overall EV	0.00097			
Trial Number × Gaze Difference	0.0015			
Residual	0.18			

Note. Improvement over no-gaze model: $\chi^2(13) = 184.7$, p < .001
