# Supplementary material for: A reinforcement learning and sequential sampling model constrained by gaze data
Source: PLoS Comput Biol. 2026 Mar 6;22(3):e1014052. doi: 10.1371/journal.pcbi.1014052 (PMC12991361; doi:10.1371/journal.pcbi.1014052)
Supplement: S12 Table — (PDF) [file pcbi.1014052.s030.pdf]

S12 Table: Logistic Mixed-Effects Model Predicting Choice Accuracy from EV Difference, Relative Value Difference, Overall EV, Overall Relative Value, and Proportional Gaze Advantage for the Correct Option in the Transfer Test of Experiment 2

**Fixed Effects**	b	SE	z	p
Intercept	1.23	0.21	5.78	< .001
EV Difference	0.27	0.10	2.57	.010
Relative Value Difference	1.20	0.16	7.62	< .001
Overall EV	-0.16	0.11	-1.41	0.16
Overall Relative Value	0.0016	0.065	0.025	0.98
Gaze Difference	0.89	0.11	7.75	< .001
**Random Effects**	Variance			
Intercept	2.088			
EV Difference	0.40			
Relative Value Difference	1.08			
Overall EV	0.55			
Overall Relative Value	0.12			
Gaze Difference	0.52			

Note. Improvement over no-gaze model: $\chi^2(7) = 540.57$, $p < .001$
