# Supplementary material for: A reinforcement learning and sequential sampling model constrained by gaze data
Source: PLoS Comput Biol. 2026 Mar 6;22(3):e1014052. doi: 10.1371/journal.pcbi.1014052 (PMC12991361; doi:10.1371/journal.pcbi.1014052)
Supplement: S13 Table — (PDF) [file pcbi.1014052.s031.pdf]

S13 Table: Linear Mixed-Effects Model Predicting log RT from EV Difference, Unsigned Relative Value Difference, Unsigned Proportional Gaze Difference, Overall Expected Value and Overall Relative Value in the Transfer Test of Experiment 2

**Fixed Effects**	b	SE	t	p
Intercept	7.024	0.051	137.41	< .001
EV Difference	-0.0030	0.0099	-0.31	0.76
Unsigned Relative Value Difference	-0.049	0.011	-4.63	< .001
Unsigned Gaze Difference	-0.080	0.013	-5.95	< .001
Overall EV	-0.011	0.012	-0.96	0.34
Overall Relative Value	-0.054	0.0084	-6.49	< .001
**Random Effects**	Variance			
Intercept	0.13			
EV Difference	0.0030			
Unsigned Relative Value Difference	0.0038			
Unsigned Gaze Difference	0.0072			
Overall EV	0.0051			
Overall Relative Value	0.0016			
Residual	0.20			

Note. Improvement over no-gaze model: $\chi^2(7) = 281.76$, $p < .001$
