# Supplementary material for: A reinforcement learning and sequential sampling model constrained by gaze data
Source: PLoS Comput Biol. 2026 Mar 6;22(3):e1014052. doi: 10.1371/journal.pcbi.1014052 (PMC12991361; doi:10.1371/journal.pcbi.1014052)
Supplement: Attachment — Submitted filename: Response to Reviewers.pdf [file pcbi.1014052.s032.pdf]

PCOMPBIOL-D-25-01886
A reinforcement learning and sequential sampling model constrained by gaze data
PLOS Computational Biology

Dear Dr. Hayes,

Thank you for submitting your manuscript to PLOS Computational Biology. After careful consideration, we feel that it has merit but does not fully meet PLOS Computational Biology's publication criteria as it currently stands. Therefore, we invite you to submit a revised version of the manuscript that addresses the points raised during the review process.

Please submit your revised manuscript by Feb 07 2026 11:59PM. If you will need more time than this to complete your revisions, please reply to this message or contact the journal office at ploscompbiol@plos.org. When you're ready to submit your revision, log on to https://www.editorialmanager.com/pcompbiol/ and select the 'Submissions Needing Revision' folder to locate your manuscript file.

Please include the following items when submitting your revised manuscript:

* A rebuttal letter that responds to each point raised by the editor and reviewer(s). You should upload this letter as a separate file labeled 'Response to Reviewers'. This file does not need to include responses to formatting updates and technical items listed in the 'Journal Requirements' section below.
* A marked-up copy of your manuscript that highlights changes made to the original version. You should upload this as a separate file labeled 'Revised Manuscript with Track Changes'.
* An unmarked version of your revised paper without tracked changes. You should upload this as a separate file labeled 'Manuscript'.

If you would like to make changes to your financial disclosure, competing interests statement, or data availability statement, please make these updates within the submission form at the time of resubmission. Guidelines for resubmitting your figure files are available below the reviewer comments at the end of this letter

We look forward to receiving your revised manuscript.

Kind regards,

Bastien Blain
Academic Editor
PLOS Computational Biology

Marieke van Vugt
Section Editor
PLOS Computational Biology

Journal Requirements:

If the reviewer comments include a recommendation to cite specific previously published works, please review and evaluate these publications to determine whether they are relevant and should be cited. There is no requirement to cite these works unless the editor has indicated otherwise.

1) We ask that a manuscript source file is provided at Revision. Please upload your manuscript file as a .doc, .docx, .rtf or .tex. If you are providing a .tex file, please upload it under the item type u2018LaTeX Source Fileu2019 and leave your .pdf version as the item type u2018Manuscriptu2019.

2) Some material included in your submission may be copyrighted. According to PLOSu2019s copyright policy, authors who use figures or other material (e.g., graphics, clipart, maps) from another author or copyright holder must demonstrate or obtain permission to publish this material under the Creative Commons Attribution 4.0 International (CC BY 4.0) License used by PLOS journals. Please closely review the details of PLOSu2019s copyright requirements here: PLOS Licenses and Copyright. If you need to request permissions from a copyright holder, you may use PLOS's Copyright Content Permission form.
Please respond directly to this email and provide any known details concerning your material's license terms and permissions required for reuse, even if you have not yet obtained copyright permissions or are unsure of your material's copyright compatibility. Once you have responded and addressed all other outstanding technical requirements, you may resubmit your manuscript within Editorial Manager.
Potential Copyright Issues:
i) Figure 1B. Please confirm whether you drew the images / clip-art within the figure panels by hand. If you did not draw the images, please provide (a) a link to the source of the images or icons and their license / terms of use; or (b) written permission from the copyright holder to publish the images or icons under our CC BY 4.0 license. Alternatively, you may replace the images with open source alternatives. See these open source resources you may use to replace images / clip-art:
- https://commons.wikimedia.org
- https://openclipart.org/.

We removed the clip-art from Figure 1B. All remaining images were drawn by hand.

Reviewers' comments:

Reviewer's Responses to Questions

Comments to the Authors:
Please note here if the review is uploaded as an attachment.

Reviewer #1: This interesting article investigates the links between gaze and choice in reinforcement learning (RL) paradigms, through the lens of sequential sampling models (SSM), specifically linear ballistic accumulator (LBA) models. The authors examine two RL experiments and compare 7 different models. They conclude that in both experiments, the model with the softmax function and additive gaze effect performs best overall.

Overall, my impression of the paper is positive. Using SSMs to better understand RL behavior seems very promising, and accounting for gaze effects is a clearly important step. It's great that there are multiple datasets, since behavior in RL tasks may vary quite a lot depending on the setup. I appreciate that the authors test several different models, that they check parameter recovery for their preferred model, and that they provide extensive analyses of the relative goodness of fits of the models in the supplements. This allows the reader to thoroughly evaluate the claims and draw their own conclusions.

<u>Author response:</u> We thank the reviewer for the encouraging and insightful feedback on our manuscript. We provide point-by-point responses to all comments below.

However, I do have some concerns about the paper, mostly regarding the modeling.
First, it strikes me as problematic to have set the theta parameter in the softmax function to 50. The optimal value of this parameter depends on the units of the values and so must surely also depend on the experiment, the participant, etc. I worry that setting this parameter to different values could affect the rankings of the sotfmax models. This concern is reinforced by the substantial difference between softmax(Q*gaze) and softmax(Q)*gaze in terms of how they explain the RT curves in Figs. S6 and S13-15. The authors should either fit theta for the model comparison exercise or demonstrate that the ranking are insensitive to a range of theta values. Once they have a preferred model, then they could fix theta to estimate the rest of the parameters, assuming that parameter recovery is negatively affected by letting theta be free.

<u>Author response:</u> Thank you for raising this important concern. We originally set the theta parameter to 50 based on a prior study that combined RL and LBA models (McDougle & Collins, 2021, *Psychon. Bull. Rev.*). The authors of that study found that theta could not be accurately recovered when left as a free parameter.

However, we agree that fixing theta could affect the rankings of the softmax models. For the revised manuscript, we re-fit the five softmax models (Models 4-8) allowing theta to be free. All figures and modeling results were updated accordingly. Fig. R1 below shows the model comparisons with theta free versus fixed in Experiments 1 and 2.

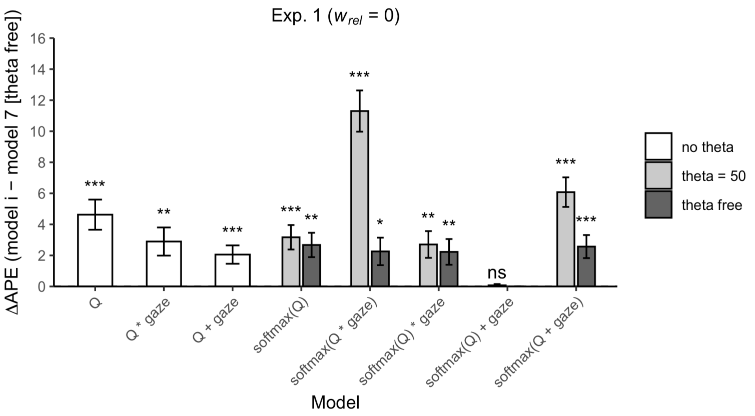

In Experiment 1, freeing theta slightly improved the fits of Models 4, 6, and 7, and dramatically improved the fits of Models 5 and 8. The model with the lowest mean APE was still Model 7 ("softmax(Q) + gaze"), but now with theta free. It was significantly favored over all other models except Model 7 with theta = 50.

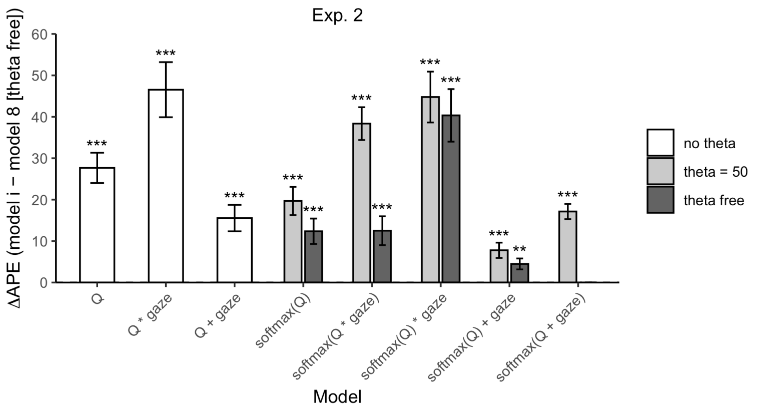

In Experiment 2, freeing theta considerably improved the performance of all softmax models. The model with the lowest mean APE is now Model 8 ("softmax(Q + gaze)"), while Model 7 was the second-best model for Experiment 2.

Fig R1. Extended model comparisons based on accumulative one-step-ahead prediction error (APE). Bars show mean APE difference scores relative to the best model. *p < .05, **p < .01, ***p < .001 (ns = not significant).

We note that although theta was not very recoverable, recovery of the other parameters was mostly unaffected by allowing theta to be free (see S4 Fig and S5 Fig).

Second, related to the previous point, I'm concerned by Figure 2A. Given that softmax(Q) + gaze is the best model in both experiments, shouldn't we expect a relatively similar ranking of the other models across the two experiments? Aside from Q + gaze being the second best model, the ranking of the other models looks uncorrelated between the experiments. This deepens my concern that the model fits may be unduly influenced by the choice of theta.

Author response: Fig. R1 above shows that the model rankings did change to some extent after freeing the theta parameter (e.g., Model 5 was particularly affected). However, even after freeing theta there were still some differences in the relative performance of the models between experiments. We do not think that this necessarily indicates a problem; rather, it could be due to differences in the nature of the choice tasks and/or the amount of data in the two experiments (Exp. 2 had nearly 4x as many observations per person). We do believe that having theta as a free parameter provides the fairest comparison, as it avoids unduly influencing any one particular model by the choice of theta.

Third, I didn't understand why the authors chose to induce competition in their model using the softmax function rather than using value difference between options. To their credit, in the Discussion they do mention that these models aren't really comparable to the DDM or aDDM, but it's unclear why they chose this modeling approach that precludes such comparisons. At the very least, this deserves more discussion, if not fitting models that have value differences in the drift rates.

Author response: Thank you for the excellent question. As we now show in S1 Appendix, the softmax is implicitly a function of Q-value differences. For example, in the two option case:

exp(theta*Q1) / [exp(theta*Q1) + exp(theta*Q2)] = 1 / [1 + exp(-theta*(Q1 - Q2))]

Accordingly, the drift rates will still be influenced by the difference between the option values. We think that the softmax may be more appropriate than the raw value difference (Q1 - Q2) for racing accumulator models like the LBA, since it also enforces non-negativity of the mean drift rates. Non-negative drift rates are important for ensuring the LBA accumulation process terminates. With the softmax, we get the best of both worlds: non-negative mean drift rates that also depend on the differences among the Q-values.

We added the following to the manuscript (lines 141-144):
"An advantage of using the softmax, as opposed to raw Q-value differences, is that it enforces non-negativity of the mean drift rates, which increases the probability that at least one accumulator will reach the decision threshold."

On a related note - why do the non-softmax models yield such flat RT curves relative to the data? Presumably this is because of the lack of competition in the model. It seems like those non-softmax models require some form of competition to have a fighting chance in these comparisons.

Author response: We agree that the lack of competition may be part of the reason for why the non-softmax models yield flat RT curves relative to the data. S1 Fig in the supplement shows that the "Q+gaze" model, for example, predicts faster RTs with increasing Q-value magnitudes, while the "softmax(Q)+gaze" and "softmax(Q+gaze)" models predict faster RTs with increasing separation between Q-values. In a typical RL task, the Q-values for the symbols within a given choice context should gradually separate with learning, but their absolute magnitudes will not always increase. This may help explain why the softmax models were better at capturing RT patterns in these tasks. Our general finding that softmax models outperformed non-softmax models suggests that some kind of competition between Q values is needed to fit choice-RT data in RL environments.

Fourth, there's very little discussion of the starting point parameters, how we should interpret them in these experiments, and how we should interpret their results.

Author response: We apologize for neglecting to mention the starting point parameters. Examining the mean parameter estimates in Fig 3, we find separation between the start point upper bound (*A*) and the decision threshold (*b*) in both experiments (Exp. 1 *b*-*A* separation: M = 453.52 [SD = 217.84]; Exp. 2 *b*-*A* separation: M = 151.37 [SD = 52.82]). When the threshold is at or near the top of the start point distribution, the model predicts "fast errors" due to start points sampled close to the threshold. With greater separation between the threshold and the start point distribution, the model predicts slower errors. Consistent with the latter, we find that incorrect choices were 401 ms slower than correct choices on average in Exp. 1, and 504 ms slower on average in the learning phase of Exp. 2 (ps < .001; paired t-tests).

We added the following to the manuscript (lines 280-284):

"Third, the average decision threshold was well above the average start point upper bound in both experiments. In the LBA model, this will lead to incorrect responses being slower than correct responses [13], which is consistent with our data: Incorrect choices were about 400-500 ms slower than correct choices, on average."

Fifth, was the Experiment 2 model fit to only the training data, or also to the transfer data? This wasn't clear from the Methods.

Author response: The models were fit to the data from both phases. We have clarified this in line 709.

Sixth, why is there no softmax(Q+gaze) model in the comparison? That seems like an obvious omission given that there are two multiplicative softmax models.

Author response: We thank the reviewer for this excellent suggestion, and we agree that a softmax(Q+gaze) model should be included in our comparisons. In our revised manuscript, we have included this model (Model 8) in both experiments, and we find that it is the best overall model for Exp. 2.

Although this model may appear to be an additive gaze model, the softmax transformation results in a multiplicative effect of gaze on mean drift rates (S1 Appendix). We therefore consider this to be a multiplicative gaze model. The fact that additive gaze best describes the data in Experiment 1 while multiplicative gaze best describes the data in Experiment 2 does complicate the story a bit; however, we think it is consistent with the mixed evidence for additive vs. multiplicative gaze in the few RL studies that have investigated this question. As we now state in the Discussion (lines 528-533):

"It is difficult to know why the evidence for additive versus multiplicative gaze was mixed across our two experiments. However, it should be noted that the two winning models provided very similar fits to the data in both experiments, and the advantage of one over the other appears to be quantitative rather than qualitative. Future studies should consider different experimental designs for which our additive and multiplicative gaze models would make qualitatively distinct predictions."

Including the softmax(Q + gaze) model increases our coverage of the space of possible models, and we believe it has made the paper stronger overall.

I also have some other questions/concerns:
Regarding the literature, the authors neglect to mention Westbrook et al. 2020, which is an important paper, as it seemingly resolves the debate between the additive and multiplicative gaze models. In particular, they argue that early in the decision gaze is multiplicative, but later, once the decision has been tentatively made, gaze becomes additive. This seems important to mention here, as the authors' modeling approach doesn't allow for a hybrid model that switches from multiplicative to additive over time. But perhaps they could include a model that has both additive and multiplicative components.

Author response: Thank you for this paper recommendation! Given that there are many potential ways to combine additive and multiplicative mechanisms in the same model, especially when combined with other modeling decisions (e.g., linear vs. nonlinear Q-drift integration), we decided to leave this intriguing task for future work. However, we agree that it would be important to mention the Westbrook et al. paper in our manuscript. We have added the following to the Discussion (lines 540-547):

"Second, additive versus multiplicative gaze is likely a false dichotomy. It could be that *both* mechanisms play a role in decision making. One study suggests that the mechanism of gaze may even change over the time course of decision formation as information accumulates toward a decision threshold. Early in a decision, gaze can amplify the influence of value information (a multiplicative effect), but later, gaze may simply correlate with the emerging choice (an additive effect) [48]. Thus, models combining both multiplicative and additive mechanisms may be useful to consider in future work."

Do the authors also have eye-tracking data during the feedback phase in Experiment 1? It would be useful to know whether participants actually look at all four stimuli during feedback. If participants only look at the target, or chosen, options, that might suggest a different model with only partial learning.

Author response: We do have eye-tracking data from the feedback phase in Experiment 1. We conducted descriptive analyses at the participant level by aggregating fixation behavior across the feedback phase. For each participant, we computed the proportion of feedback trials on which they fixated at least once on all symbols, only the chosen symbol, or no symbols. All participants fixated on at least one symbol on 100% of feedback trials. When averaging across all participants, fixation of all symbols occurred on 23.0% of trials and fixation restricted to the chosen symbol occurred on 9.2% of trials. This suggests that learning was not restricted to the target option alone. Mean fixation durations were longer for the available but unchosen options (M = 273.99 ms) than the chosen (M = 242.94 ms) and unavailable options (M = 242.74 ms) , $F(1.98, 122.62) = 20.20$, $p < .001$, $n^2_p = 0.25$.

In Experiment 2, we conducted the same descriptive analyses on eye movements by focusing on fixations to the outcomes. Averaged across participants, both outcomes were fixated on 61.8% of feedback trials and fixation was restricted to the chosen outcome on 14.1% of trials. However, on 17.0% of trials, participants did not fixate on either outcome during feedback. These results indicate that participants typically sampled both outcomes during feedback, but some participants exhibited disengagement from the options values on some trials. Like in the first experiment, this suggests that learning was not restricted to the target option alone.

Do the authors have an explanation for why gaze has a larger effect on choice in Experiment 1?

Author response: We believe the reviewer is referring to the following statement we made in the original manuscript based on a comparison of the estimated beta_gaze parameters:

“It is interesting that [beta_gaze] estimates were higher on average in Experiment 1, which might suggest that gaze has a stronger influence on choice in more complex environments.”

We have removed this sentence from the revised manuscript because this result appears to depend on the particular model used to describe the data. It is true that the estimates of the gaze parameter were higher in Exp. 1 using the softmax(Q) + gaze model, but when we use the softmax(Q + gaze) model, which was best for Exp. 2, we find that the gaze parameter was higher on average in Exp. 2 (see Fig. 3). The gaze parameter does function slightly differently in these two models (S1 Appendix), and therefore it probably does not make sense to compare estimates between models. For this reason, and because we did not design the two experiments to be directly comparable, we decided to remove this statement.

I think the authors are somewhat burying the lede by not making a bigger deal about the binomial distribution of relative vs. absolute value representation. It seems like that is an important finding and should be mentioned in the abstract.

Author response: Thank you for the suggestion; we agree. The revised abstract now reads:

“Further, we show that [the model] captures a variety of empirical effects, including gaze biases on choice and response time, as well as individual differences in absolute versus relative valuation.”

It seems worth noting that in the aDDM, faster RT across trials and for N++ trials (and slow N— trials) would be automatically explained by the multiplicative gaze effects, as the Q values are higher in those cases and the aDDM predicts faster decisions for higher overall value.

Author response: Thank you for this suggestion. We have added the following on lines 349-351:

“We would expect the aDDM, which predicts faster decisions for higher overall value [22], to also produce faster RTs on Neutral++ trials and slower RTs on Neutral-- trials.”

In the results on pages 10 and 11, what effect does overall value have on choice and RT?

Author response: Overall value (the summed EVs of the available symbols) was included as a control predictor in our mixed effects regression models to test whether gaze would predict behavior over and above the other predictors. Overall value did not have an effect on choice (S8, S9, and S12 Tables). However, for RT, the effect of overall value is slightly different. In Experiment 1, higher overall values were associated with faster response times (S10 Table). In Experiment 2, higher overall *relative* values, but not absolute expected values, were associated with faster response times in the transfer test (S13 Table). The latter result is reflected in the faster mean RTs for N++ trials, as these are trials in which both options had high relative value. Taken together, these results suggest that while overall value does not influence choice accuracy, it may influence how quickly people respond.

Do the authors have an explanation for why the trial interaction is different between the two experiments?

Author response: We do not have a solid explanation for the differences between experiments involving trial interactions. We can speculate that it may have to do with the number of symbols shown on the screen. Experiment 1’s inclusion of four symbols at a given time on the screen may have changed how gaze was distributed across trials as participants learned multiple option values in a given context. As learning progressed in this experiment, gaze differences may have significantly decreased and reduced the influence of gaze on choice over time. However, in Experiment 2, only two symbols were presented at a time, which may have limited variability in gaze across trials and contributed to the nonsignificant negative interaction. Similar reasoning could apply to the changes in the effect of gaze on RTs across trials. While we think these interactions are interesting and worth exploring in future work, our primary goal was to show that gaze affects choice and RT over and above trial number and option values.

The fitted non-decision times are substantially shorter than we normally see in other studies. This seems worth commenting on.

Author response: Thank you for raising this concern. We hesitate to over-interpret the non-decision time estimates, as this parameter was not highly recoverable ($0.50 < r < 0.82$; see S4 and S5 Figs.). We note that a prior study using a similar model had difficulty estimating non-decision time, and had to fix it to a constant (150 ms; McDougle & Collins, 2021, *Psychon. Bull. Rev.*). They noted that when they fit non-decision time as a free parameter, it “tended to take on values well below biologically reasonable minimum human RTs (i.e., < 100 ms)” (p. 27). While most of the non-decision time estimates in our first experiment were above this threshold, several in the second experiment fell below 100 ms (Fig. 3). Therefore, we would encourage caution when estimating this parameter. Thankfully, it is not a parameter that was of primary interest in our study.

We have added the following in lines 278-280:
“However, it should be noted that in Experiment 2, several non-decision time estimates were below biologically plausible minimum RTs for humans (< 100 ms; [28]), and therefore should be interpreted with caution.”

In the declining threshold model, is delta significantly different from 0?

Author response: Thank you for this question. The mean delta parameter from the softmax(Q + gaze) model with declining threshold was significantly above zero, $t(49) = 3.78$, $p < .001$. We added this information to S2 Appendix.

The authors may want to comment on Fig S12, namely the fact that the non-softmax models seem to do a better job of capturing the RT changes for N++ and N- -.

Author response: Thank you for this suggestion. We have added the following on lines 354-356:

"Although none of our models perfectly captured the pattern of mean RTs across the transfer choice categories, this is one aspect of the data that the linear integration models seem to capture better than the softmax models (S12 Fig)."

References:

Westbrook, A., Van Den Bosch, R., Määttä, J. I., Hofmans, L., Papadopetraki, D., Cools, R., & Frank, M. J. (2020). Dopamine promotes cognitive effort by biasing the benefits versus costs of cognitive work. Science, 367(6484), 1362-1366.

Reviewer #2: This is a very good manuscript, clearly and thoroughly presenting a well-designed study. The study shows how visual attention to choice options impacts the learning (or perhaps elucidating) of the values of the options, in addition to the previously-reported impact on the choices themselves. The authors present a computational model that includes elements of reinforcement learning, sequential sampling, and gaze bias, and their model successfully accounts for a variety of experimental findings. I think this paper is already well along the way towards being publishable. I list below some minor suggestions and questions for the authors to consider:

Author response: We thank the reviewer for their encouraging feedback! Below, we provide point-by-point responses to each suggestion and question.

lines 141-145: Perhaps the reader would benefit from a bit of an explanation of this, specifically how the softmax rule makes the decision variables dependent on each other and thus the racing accumulators are not independent.

Author response: Thank you for this suggestion; we agree that this could use more explanation. In short, it occurs due to the mathematics of the softmax. We now show in S1 Appendix that the softmax is implicitly a function of Q-value differences. For example, in the two option case:

exp(theta*Q1) / [exp(theta*Q1) + exp(theta*Q2)] = 1 / [1 + exp(-theta*(Q1 - Q2))]

We also reworded the section in the main text as follows (lines 139-151):

"The softmax causes the mean drift rates to depend on the *differences* between Q-values, similar to models that accumulate the relative evidence for choosing one option over another (e.g., the aDDM [14]; see S1 Appendix). An advantage of using the softmax, as opposed to raw Q-value differences, is that it enforces non-negativity of the mean drift rates, which increases the probability that at least one accumulator will reach the decision threshold. With appropriate parameter settings, the softmax function can be very sensitive to small differences in the underlying Q-values, producing sharper decision boundaries compared to linear integration models (S1 Fig, left panels). The softmax also causes predicted response times to depend only on the difference between Q-values, with longer RTs for difficult choices (i.e., small Q-value differences). Linear integration models instead predict a magnitude effect: RTs are longer when both Q-values are small and faster when both Q-values are large (S1 Fig, right panels)."

255-257: The authors should note that it comparing drift scalar components should be done in a relative way, not absolute. Thus the statement should perhaps be about gaze having a stronger influence on choice *relative* to the influence of learned values.

Author response: Thank you for raising this point; we completely agree that comparing drift scalar components should be done in a relative way. We ended up removing the sentence in question ("It is interesting that [beta_gaze] estimates were higher on average in Experiment 1, which might suggest that gaze has a stronger influence on choice in more complex environments.") because after further analysis, it appears that particular result depends on the model being used to describe the data.

377-379: The authors show that gaze difference has an effect on choice even after controlling for other factors, such as value difference. However, they do so by including all the factors as co-regressors in the same regression model. A stricter way of controlling for the influence of one variable would be to orthogonalize the second variable with respect to the first (e.g., regressing one on the other and taking

the residuals as the second regressor in the model of interest). Did the authors try that, and if so, did it change any of the results?

Author response: This is an interesting question. We did not try to do this. However, we believe that including all factors as co-regressors in the same regression model is an equally effective way to test whether gaze can predict choice over and above (i.e., controlling for) other factors, such as value difference. Essentially, with this approach we can ask, "If a person were choosing between two options with equal expected values, but one option received relatively more gaze, would they be more likely to choose the option with the gaze advantage?" The coefficient for gaze represents the estimated effect of gaze after partialling out the effects of all other regressors.

There are two reasons why we did not try the two-step approach. First, computing residuals in a mixed effects logistic regression is nontrivial due to the binary nature of the DV and the multilevel structure of the model. Second, in ordinary least-squares regression, the two-step approach and the simultaneous approach give identical results for the target predictor (e.g., gaze), as can be seen in the following R code example. We would expect the results to be very similar in this case as well.

```
set.seed(12345)

# Correlated regressors X1 and X2
X <- MASS::mvrnorm(n=100, mu=c(0,0), Sigma=matrix(c(1,.5,.5,1), nrow=2))

# Y is a linear function of X1 (not X2)
Y <- 2 + 1*X[,1] + 0*X[,2] + rnorm(n=100)

# X2 and Y are spuriously related when X1 is not controlled for
summary(lm(Y ~ X[,2]))

# 1. Simultaneous regression: X2 no longer significant
summary(lm(Y ~ X))

# 2. Two-step regression. First, residualize X2 on X1.
X2.resid <- residuals(lm(X[,2] ~ X[,1]))

# Then add residualized X2 as a second regressor. Results for X2 are identical.
summary(lm(Y ~ X[,1] + X2.resid))
```

507-509: I wonder if allowing the softmax inverse temperature parameter to be free would be one way of interpolating between a linear mapping and the softmax mapping that the authors used (with a fixed inverse temperature).

Author response: Thank you for the excellent suggestion. In our revised manuscript we re-fit the five softmax models (Models 4-8) with theta as a free parameter. All figures and modeling results were updated accordingly. The mean estimates of theta in both experiments were well below the value of 50 that we fixed it to in our original manuscript (see Fig 3). The lower values of theta indicate that mean drift rates were not quite as sensitive to small Q-value differences as our original models may have suggested.

Reviewer #3: The manuscript introduces a reinforcement-learning sequential-sampling model (RL-SSM) that integrates eye-tracking data to predict choices and response times in repeated decision tasks. The model assumes that subjective values (Q-values) learned through trial-and-error and proportional gaze independently contribute to drift rate in the sequential sampling process. Two experiments evaluate the model against alternatives that differ in how gaze and values are combined. The authors find that a softmax-based value mapping with an additive gaze term provides the best out-of-sample predictions. The model captures a wide range of behavioral and gaze-related effects, and the work addresses an important gap in the literature by combining learning and gaze-influenced decision dynamics within a computationally tractable model.

Overall, the manuscript presents a valuable integration of gaze into RL-SSMs and offers compelling empirical demonstrations. The approach is promising and clearly articulated. Several points would, however, benefit from clarification or extension to strengthen the interpretation and robustness of the findings.

Author response: We thank the reviewer for their encouraging assessment of our manuscript and constructive feedback! We provide point-by-point responses to each comment below.

Major Comments

1. The model comparison relies exclusively on APE, which, as I understand it, does not penalize model complexity. Since the winning model is also the most flexible, a complexity-adjusted comparison or a brief discussion of how complexity affects APE would help ensure that the conclusions are not driven by differences in the number of free parameters.

Author response: We share the reviewer's concern about the importance of accounting for model complexity, not just goodness of fit. APE does account for complexity. As Wagenmakers, Grünwald, and Steyvers (2006) explain, "By measuring predictive performance for unseen data, the APE method automatically adjusts for model complexity in a conceptually straightforward manner" (p. 154), and APE "automatically incorporates a penalty for model complexity" (p. 164). The advantage of APE over methods like AIC and BIC is that it is sensitive to the functional form of the model parameters (i.e., how they function together in the model)--not just to the number of parameters. Thus, APE adjusts for complexity in a more comprehensive way than AIC or BIC.

We added the following on lines 213-215:

"Further, because it evaluates predictions on unseen data, APE adjusts for model complexity in a way that depends on a model's functional form, not just the number of parameters."

2. In Experiment 1, fixing the relative-encoding parameter ω_rel to 0 is understandable given identifiability constraints, but it is not clear why absolute encoding is preferred over fully relative encoding. Since many participants in Experiment 2 show strong relative valuation, comparing fixed ω_rel=0 versus ω_rel=1, or providing a principled justification for choosing ω_rel=0, would help validate this modeling decision.

Author response: This is an excellent suggestion. We agree that it is important to validate these kinds of modeling decisions. Since many participants in Experiment 2 showed strong relative valuation, there is no compelling reason to prefer fully absolute encoding over fully relative encoding for the models in Experiment 1. We therefore re-fit the models in Experiment 1 with the constraint w_rel = 1 and present the results in Fig. R2 below. The results are nearly identical for w_rel = 0 and w_rel = 1. Regardless of whether we assume fully absolute or fully relative encoding, the softmax(Q) + gaze model is the best

model on average in Experiment 1. We now state on lines 196-197: “however, the conclusions are the same if we instead fix w_rel to 1 (relative encoding).”

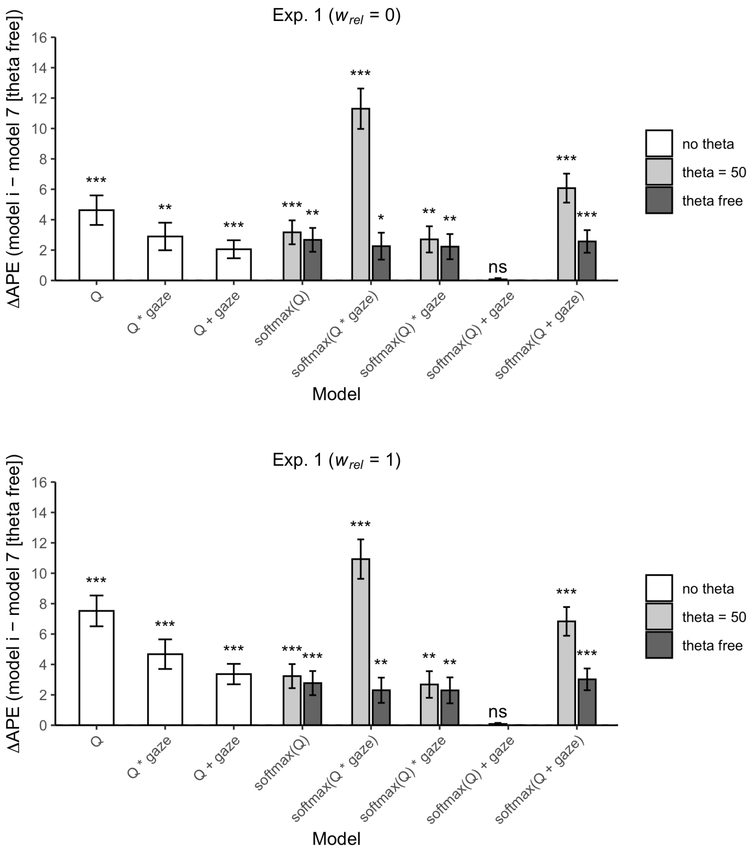

Fig. R2. Extended model comparisons testing the effect of fixing the relative encoding parameter to 0 (fully absolute encoding) or 1 (fully relative encoding) in the first experiment. Bars show mean APE difference scores relative to the best model. *p < .05, **p < .01, ***p < .001 (ns = not significant).

3. [238-248] The trial-by-trial example in Fig. 2B is helpful for illustrating how gaze can improve predictions on erroneous trials. However, since this point is later referenced in a more general way in the discussion, a brief group-level summary of how often the gaze-augmented model improves error prediction relative to the no-gaze model would help clarify whether the effect is systematic across participants or limited to selected cases.

Author response: Thank you for this suggestion. Fig. 2C in the revised manuscript now shows mean choice prediction accuracy for all models as a function of whether the participant made a correct or incorrect choice, and whether they gazed longer at the correct or incorrect symbol. The data were averaged across all participants. We find that the gaze-augmented models were more accurate than the no-gaze models on trials where participants looked longer at the incorrect symbol and subsequently selected it.

We added the following paragraph to the manuscript (lines 245-267):

“Fig 2B shows how accurate each model was at predicting choice, averaging across participants. Models constrained by gaze data (dark gray bars) predicted choice more accurately, on average, than models without gaze data (light gray bars). This was especially true in Experiment 1, where the gaze models achieved nearly 6-10% higher mean accuracy than the no-gaze models. Fig 2C provides more detail, breaking down model prediction accuracy into four types of trials based on whether the participant made a correct (maximizing) or incorrect (non-maximizing) choice, and whether they gazed longer at the correct or incorrect symbol before choosing. Models constrained by gaze data predicted choice more accurately, on average, when the participant chose the symbol they looked at longer, which characterized about 65-70% of all trials. Models without gaze, in contrast, were more accurate when the participant made a correct choice, regardless of which symbol they looked at longer. The advantage of the gaze models is especially clear on trials where the participant looked longer at the incorrect symbol and subsequently selected it: Most of the gaze models predicted these erroneous choices better than chance (.50), whereas the no-gaze models were well below chance (see S3 Fig for an example of how one of the gaze models predicted an individual participant's choices on a trial-by-trial basis). Lastly, Fig 2C suggests that the multiplicative gaze models (Models 2, 5, 6, and 8) were more strongly affected by gaze than the additive gaze models (Models 3 and 7). In particular, multiplicative models were more accurate than additive models on trials where the participant looked longer at the incorrect symbol and subsequently chose it, but they were less accurate than additive models on trials where the participant looked longer at the incorrect symbol before choosing the correct symbol.”

The original Fig. 2B, which showed model predictions for a single participant, has been moved to the supplement (S3 Fig).

4. [253-257] The discussion of β_Q and β_gaze relies on visual inspection of their empirical distributions. If claims about positivity or parameter differences across experiments are intended to be inferential, providing confidence intervals or statistical tests would avoid possible overinterpretation.

Author response: We agree that providing statistical tests would allow us to make stronger claims about the positivity of these parameters. We added the following (lines 272-274):

“The means of these parameters were significantly greater than zero in both experiments and in both models ($ps < .001$; one-sample t-tests).”

5. [370-374] In the mixed-effects analyses of accuracy and RT, the model uses fixed task-defined EVs as predictors. Because these EVs do not reflect learning-induced changes in subjective value differences, I am not sure I fully understand how this specification controls for learning effects when estimating the additional contribution of gaze. Clarifying this rationale, or considering learned Q-value differences, would strengthen the argument.

Author response: Thank you for this suggestion. We agree that fixed EVs do not, by themselves, reflect learning-induced changes in subjective value representations. For that reason, we included trial number and its interactions with EV-based predictors to capture learning over time. Specifically, trial number captures the general improvement in accuracy over time that occurs with learning, and the trial-by-EV-difference interaction captures how the influence of value-related difficulty can change as learning progresses. We considered including learned Q-values as predictors. However, the estimated Q-values depend on the chosen model and on the participant’s parameters (e.g., learning rate). For these reasons, we decided to use trial number and its interaction with task-defined EV differences to test whether gaze

predicts accuracy and RT over and above these factors.

6. A trial-dependent decreasing decision threshold improves RT fits (S2 Appendix), yet this model is not included in the formal model comparison. A brief explanation of why it was not evaluated with APE would clarify whether this decision reflects theoretical priorities, identifiability constraints, or practical considerations.

Author response: Thank you for this suggestion. The inclusion of decreasing thresholds was done after the fact to show how the model's fit to the aggregate RT curves could be improved. Because these models were not originally planned, and because closely fitting the aggregate RT curve was not a theoretical priority, we did not formally evaluate these models with APE. We see this as a promising direction for future research.

We have added the following sentence (lines 310-312): "Since [closely fitting the RT curves] was not a theoretical priority in the current study, we chose not to formally evaluate the decreasing threshold models with APE."

7. [519-526] The discussion proposes future extensions in which models would predict gaze based on learned values and other factors (such as salience or uncertainty). In learning tasks, however, gaze may also influence value updating itself. Acknowledging this reciprocal influence would provide a more complete perspective on potential extensions of the modeling framework.

Author response: Thank you for this suggestion. We agree that acknowledging the reciprocal influence of gaze on value updating is important for RL tasks. We are currently expanding on this idea in a follow up project, but it is beyond the scope of the present paper to address it in depth.

We have added the following sentence to the Discussion as a direction for future research (lines 584-586):

"It is also possible that gaze may play a role in value updating, as attention during the feedback phase could influence subsequent value representations [8]."

Minor

a. Fixing θ enhances identifiability, but the specific value of 50 is only briefly justified. Since θ strongly affects drift mapping, a brief sensitivity analysis or additional justification would increase confidence that results do not hinge on this value.

Author response: Thank you for the excellent suggestion. Given that this concern was raised by the other reviewers as well, in our revised manuscript we re-fit the five softmax models (Models 4-8) with theta as a free parameter. All figures and modeling results were updated accordingly. Although theta was not particularly recoverable, recovery of the other parameters was mostly unaffected (S4 Fig and S5 Fig). Model comparison results with and without theta as a free parameter can be found in our response to Reviewer 1's first comment.

b. [139-140] The text states that the steep softmax "produces greater overall accuracy (S1 Fig.)," but S1 Fig. does not present accuracy results. Adjusting the citation or wording would help readers follow the argument more easily.

Author response: We adjusted the wording on lines 144-147 to better align with S1 Fig:

"With appropriate parameter settings, the softmax function can be very sensitive to small differences in the underlying Q-values, producing sharper decision boundaries compared to linear integration models (S1 Fig, left panels)."

c. [118] In the notation "Uniform(0,A)", A is not defined until line 190.

Author response: We added the following on lines 119-120: "with lower bound 0 and upper bound *A*."

d. The main model line is described as "maroon" through the manuscript (e.g. [269]) but to me it appears more purple.

Author response: We agree! R calls that color "maroon" even though it seems more purple to us as well. We have changed it to "purple" throughout the manuscript.

e. I did not find citations to Figures S10 and S11 or to Tables S6 to S13 in the main text.

Author response: Thank you for catching this. We now include references to Figures S10 and S11 on line 314 and to Tables S6 and S13 in lines 400 and 706.

Have the authors made all data and (if applicable) computational code underlying the findings in their manuscript fully available?
The PLOS Data policy requires authors to make all data and code underlying the findings described in their manuscript fully available without restriction, with rare exception (please refer to the Data Availability Statement in the manuscript PDF file). The data and code should be provided as part of the manuscript or its supporting information, or deposited to a public repository. For example, in addition to summary statistics, the data points behind means, medians and variance measures should be available. If there are restrictions on publicly sharing data or code —e.g. participant privacy or use of data from a third party—those must be specified.

Reviewer #1: Yes

Reviewer #2: Yes

Reviewer #3: **No:** I don't know if it's available. It's not specified in the manuscript.

PLOS authors have the option to publish the peer review history of their article (what does this mean?). If published, this will include your full peer review and any attached files.

If you choose “no”, your identity will remain anonymous but your review may still be made public.

Do you want your identity to be public for this peer review? For information about this choice, including consent withdrawal, please see our Privacy Policy.

Reviewer #1: No

Reviewer #2: No

Reviewer #3: No

Figure resubmission:

While revising your submission, we strongly recommend that you use PLOS’s NAAS tool (https://ngplosjournals.pagemajik.ai/artanalysis) to test your figure files. NAAS can convert your figure files to the TIFF file type and meet basic requirements (such as print size, resolution), or provide you with a report on issues that do not meet our requirements and that NAAS cannot fix.

After uploading your figures to PLOS’s NAAS tool - https://ngplosjournals.pagemajik.ai/artanalysis, NAAS will process the files provided and display the results in the "Uploaded Files" section of the page as the processing is complete. If the uploaded figures meet our requirements (or NAAS is able to fix the files to meet our requirements), the figure will be marked as "fixed" above. If NAAS is unable to fix the files, a red "failed" label will appear above. When NAAS has confirmed that the figure files meet our requirements, please download the file via the download option, and include these NAAS processed figure files when submitting your revised manuscript

Reproducibility:

To enhance the reproducibility of your results, we recommend that authors of applicable studies deposit laboratory protocols in protocols.io, where a protocol can be assigned its own identifier (DOI) such that it can be cited independently in the future. Additionally, PLOS ONE offers an option to publish peer-reviewed clinical study protocols. Read more information on sharing protocols at https://plos.org/protocols?utm_medium=editorial-email&utm_source=authorletters&utm_campaign=protocols
